# Island Biogeography in the Anthropocene: Natural and Anthropogenic Drivers of Plant Diversity in the Miaodao Archipelago

**DOI:** 10.1002/ece3.72329

**Published:** 2025-10-17

**Authors:** Haitao Yu, Xue Feng, Yuhuang Lin, Ying Yang, Zixiong Song, Shie Ching Ang, Qingchun Wang

**Affiliations:** ^1^ School of Ecology and Nature Conservation Beijing Forestry University Beijing China

**Keywords:** human interference, Island biogeography, Miaodao archipelago, plant diversity, spatial pattern

## Abstract

Island ecosystems are particularly sensitive to natural and anthropogenic environmental changes, and island plant communities undergo restructuring driven by multiple interacting factors. However, how these factors collectively influence species diversity and community composition on temperate inhabited islands remains unclear. This study assessed changes in vascular plant diversity across spatial gradients of area, isolation, climate, and human disturbance on 10 inhabited islands in Miaodao Archipelago. Based on comprehensive field surveys, we recorded 485 plant taxa and analyzed alpha and beta diversity patterns for different life forms (trees, shrubs, herbs, and invasive plants) using general linear regression, generalized linear models, redundancy analysis, and Mantel tests to identify the primary drivers. We found that island area is the most important predictor of species richness and species distribution. Contrary to classical island biogeography expectations, isolation (distance from the mainland or nearest island) has limited effects on richness, especially under conditions of strong human disturbance. The annual mean wind speed has a positive effect on shrub and herb richness but reduces community diversity and evenness. The human influence index significantly disrupted community structure but did not affect species richness. Beta diversity analysis showed that all plants and trees were driven by both ecological niche differentiation and dispersal processes, while herbs were primarily constrained by dispersal. Our findings indicate that plant communities on temperate inhabited islands are shaped by both natural gradients and human activities, with different plant life forms exhibiting distinct responses. These discoveries underscore the necessity of prioritizing the protection of larger islands and managing human disturbance to maintain biodiversity in coastal archipelagos in the context of global change.

## Graphical abstract

This study assessed changes in vascular plant diversity across spatial gradients of area, isolation, climate, and human disturbance on 10 inhabited islands in Miaodao Archipelago. We found that island area is the most important predictor of species richness and species distribution. Annual mean wind speed has a positive effect on shrub and herb richness but reduces community diversity and evenness. The human influence index significantly disrupted community structure. Beta diversity analysis showed that all plants and trees were driven by both ecological niche differentiation and dispersal processes, while herbs were primarily constrained by dispersal.

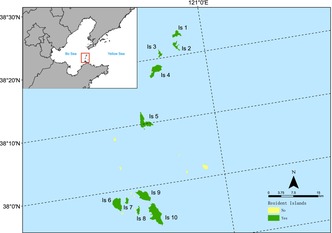

## 1 Introduction

Island ecosystems have long been regarded as natural laboratories for studying the mechanisms of biodiversity formation and maintenance due to their unique geographical isolation, limited resources, and habitat heterogeneity (Russell and Kueffer 2019; Matthews and Triantis 2021). The theory of island biogeography (Macarthur and Wilson 2001) proposes that the species richness of islands is determined by the balance between extinction and immigration rates, which are closely related to island area and isolation, i.e., richness is positively correlated with area and richness is negatively correlated with isolation. Many previous studies have shown that both island area (Liu et al. 2023; Hannus and Von Numers 2008; Li et al. 2025; Mologni et al. 2021) and isolation (Liu et al. 2023; Xu et al. 2023; Cabral et al. 2014; Fahrig 2013) significantly influence plant species richness. Besides, precipitation (Valli et al. 2019; Wang et al. 2019; Kreft et al. 2008), temperature (Valli et al. 2019; Wang et al. 2019; Kubota et al. 2015), wind speed (Irl et al. 2021; Xie et al. 2024), human activities (Helmus et al. 2014; Gleditsch et al. 2023; Matthews and Triantis 2021; Chepinoga et al. 2012), and landscape (Schrader et al. 2020; Sfenthourakis and Triantis 2009) also affect island plant richness. Investigating how environmental factors influence species richness and understanding the geographical distribution and dynamics of species diversity can significantly contribute to theoretical foundations for species conservation.

Analyzing species diversity at different levels has always been a hot spot in ecological research. Beta diversity reflects the differences in species composition at different spatial and temporal scales or environmental gradients (Whittaker 1960), and the understanding of its spatial and temporal patterns is of great significance in guiding the design of nature reserves and the conservation of regional species diversity (Socolar et al. 2016; Jacquemyn et al. 2007). It is currently believed that beta diversity is mainly shaped by the combined influence of diffusion processes and ecological niche differentiation (Legendre et al. 2009; Jiang et al. 2021). The diffusion process that beta diversity depends on the degree of isolation between communities or regions and the dispersal ability of biological groups; the lower the degree of isolation or the stronger the dispersal ability of species, the lower the beta diversity (Catano et al. 2017; Fattorini 2010). The ecological niche differentiation indicates that species are adapted to specific environments to generate a specialized ecological niche, and the more similar the environments are, the lower the beta diversity is (Whittaker 1960; Xing and He 2019). Therefore, examining the relative importance of these two processes in driving the formation of beta diversity in different taxa remains one of the research hotspots in the field of biodiversity.

Plant functional types, such as life forms, may also significantly influence plant diversity (Ewers and Didham 2006; Hu et al. 2019), which are strong proxies for differences in life‐history strategies, dispersal abilities, ecological adaptations, and habitat requirements (Schrader et al. 2020; Wullschleger et al. 2014; Rodríguez‐Loinaz et al. 2012). Herbaceous plants are smaller, have shorter life cycles, show more diversified environmental adaptations, and are more likely to colonize resource‐poor islands (Schrader et al. 2020; Pierce et al. 2017). Trees and shrubs, which typically follow life‐history strategies associated with longevity, demographic stability, and resource acquisition, are susceptible to environmental filtering and anthropogenic disturbance and are more likely to survive on resource‐stabilized islands (Díaz et al. 2016; Šímová et al. 2018; Schrader et al. 2020). In addition, similarity distance decay was much shallower for herbaceous plant assemblages on islands than for shrub or tree assemblages, which may be related to the higher dispersal capacity of herbaceous plants (König et al. 2017; Thomson et al. 2010). Therefore, it is still not clear whether the diversity of different species in life forms responds to environmental factors in the same way.

Miaodao Archipelago is located in the Bohai Strait and is an ecological hub connecting the Jiaodong Peninsula and the Liaodong Peninsula. It has a variety of terrain and vegetation types (Chi et al. 2016). As a typical inhabited island group in North China, it has a wide range of area, isolation, climate, and human disturbance gradients, making it an ideal area for studying the pattern of island plant diversity and environmental driving factors (Chi et al. 2018; Chi, Xie, and Wang 2019). Previous studies on plant diversity in the Miaodao Archipelago were often limited by a small number of surveyed islands and rarely integrated the combined effects of island attributes, climate, landscape structure, and human activities. Moreover, they seldom explored the differential responses of various plant life forms to environmental factors, nor did they consider the role of invasive species in shaping island biodiversity (Chi et al. 2016; Chi, Sun, et al. 2019; Qi et al. 2015; Chi et al. 2025). In addition, most studies on island plant diversity have focused primarily on verifying species richness, with incomplete assessments of diversity. In contrast, this study focused on 10 inhabited islands in the Miaodao Archipelago. It incorporated the Shannon–Wiener, Simpson, and Pielou indices to comprehensively consider species richness, evenness, and dominance, thereby providing a more comprehensive assessment of diversity (Chen et al. 2024; Ma et al. 2023). Based on detailed island‐scale inventory data, we classified species into trees, shrubs, and herbs, and examined their relationships with eight environmental variables spanning physical, climatic, and anthropogenic dimensions. Importantly, this study is among the first to investigate the differential environmental responses of plant life forms on islands and to incorporate invasive plant species into the analysis. This dual focus not only advances our understanding of community assembly mechanisms in fragmented island habitats but also provides practical implications for managing biological invasions in ecologically sensitive regions. Our integrative approach parallels recent advances in mountain biodiversity research (Bisht et al. 2022), where multi‐factor analyses have proven effective in disentangling complex ecological patterns.

As a result, from a multidimensional perspective, this study proposes to address the following questions: (1) What are the characteristics of species composition and distribution of vascular plants in Miaodao Archipelago? Are there differences between plants of different life forms? (2) Is island biogeography theory applicable to inhabited islands? What are the different effects of environmental factors on the diversity of plants of different life forms? (3) Under the combined influence of multiple environmental factors, what are the most critical driving factors affecting island plant diversity? Are the driving factors of plants of different life forms the same?

## 2 Materials and Methods

### 2.1 Study Area

Miaodao Archipelago, located in the Bohai Strait, comprises 32 islands (including 10 permanently inhabited islands), with a total area of about 56 km^2^. The geographical coordinates are 120°36′–120°56′ east longitude and 37°53′–38°23′ north latitude, located between the Jiaodong Peninsula and the Liaodong Peninsula.

Miaodao Archipelago is located in the transition zone between the Eurasian continent and the North China Plain. It belongs to the continental climate of the eastern Asian monsoon zone, with a mild and humid climate, and is characterized by warm winters and cool summers. The annual average temperature is 12.0°C, and rainfall is 537 mm. The Miaodao Archipelago has a diverse terrain, including hills, plains, coasts, mountains, etc. There are more than 40 hills over 100 m, and the highest altitude is 202.8 m (Chi et al. 2018). The forest type is mainly artificial forest, including black pine forest, red pine forest, locust forest, oak forest, etc., with a forest coverage rate of 56% (Chi et al. 2016).

**FIGURE 1:**
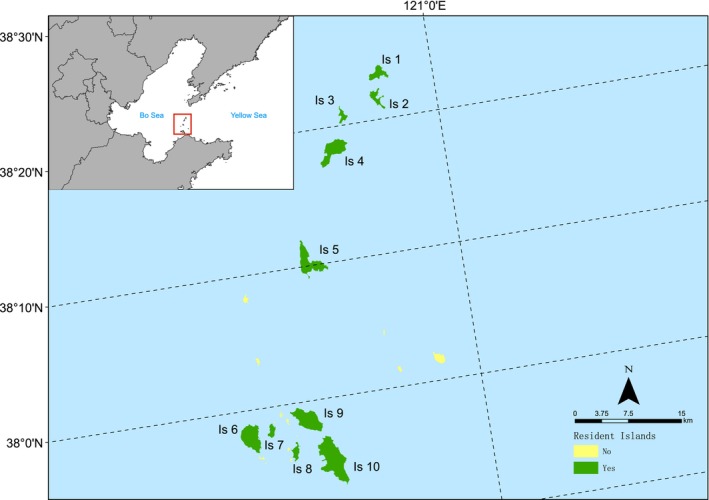
Geographical location of the 10 inhabited islands. Is 1: Beihuangcheng Island; Is 2: Nanhuangcheng Island; Is 3: Xiaoqin Island; Is 4: Daqin Island; Is 5: Tuoji Island; Is 6: Daheishan Island; Is 7: Xiaoheishan Island; Is 8: Miaodao Island; Is 9: Beichangshan Island; Is 10: Nanchangshan Island.

This study selected 10 inhabited islands as the research area (Figure 1), including Beihuangcheng Island, Nanhuangcheng Island, Tuoji Island, Daqin Island, Xiaoqin Island, Daheishan Island, Xiaoheishan Island, Miaodao Island, Beichangshan Island, and Nanchangshan Island. The islands are relatively disturbed by human activities. They are typically inhabited islands and temperate coastal islands in North China, with an area range of 1.21–14.28 km^2^ and a distance range of 6.89–50.92 km from the mainland coast (Table 1).

**TABLE 1:** Island environmental factors (A, area; AMW, annual mean wind; AP, annual precipitation; DM, distance to the mainland; DN, distance to the nearest island; HII, human influence index; PD, patch density).

Island name	A	DM	DN	HII	AP	AMW	PD
Beihuangcheng Island	2.68	41.03	1.22	22.75	634.36	4.69	170.57
Nanhuangcheng Island	1.93	44.10	1.22	25.72	635.87	4.68	203.39
Daqin Island	6.52	50.92	2.16	17.56	654.96	4.74	199.24
Xiaoqin Island	1.21	48.38	2.16	15.82	662.30	4.60	179.25
Daheishan Island	7.50	16.21	0.48	19.03	651.84	4.66	129.95
Xiaoheishan Island	1.27	16.84	0.31	20.62	661.95	4.64	170.49
Beichangshan Island	8.10	14.83	0.82	35.31	680.58	4.79	158.45
Nanchangshan Island	14.28	6.89	0.88	42.93	713.74	4.71	177.48
Tuoji Island	7.21	35.82	8.48	16.21	685.91	4.68	204.15
Miaodao Island	1.49	12.29	0.04	21.90	698.00	4.60	211.45

### 2.2 Species Data

A survey of vascular plant species was conducted on 10 inhabited islands from June 20 to July 20, 2023, with an average interval of 3 days between surveys on each island. The survey employed a combination of transects and plot‐based sampling. Transects were established using a circular island transect approach, with both east–west and north–south lines laid out through the central part of each island. All vascular plant species occurring within 5 m on either side of each transect were recorded (Xie et al. 2023). A total of 118 transects were established across the 10 islands.

To ensure comprehensive species documentation, sample plots were established to represent different types of plant communities using standard ecological sampling methods. Following the principle of uniform spatial distribution, and taking into account island area, vegetation representativeness, and topographic variation, 20 m × 20 m forest plots were randomly established. A total of 40 forest plots were established across the 10 islands (Table S1). Within each forest plot, five 2 m × 2 m shrub‐layer subplots were set at the four corners and the center, and ten 1 m × 1 m herb‐layer subplots were established, with nine placed in fixed positions and one selected randomly. In each forest plot, the following data were recorded: for the tree layer (DBH ≥ 4 cm), species name, diameter at breast height (DBH), height, crown width, growth condition, and other relevant information; for the shrub layer (DBH < 4 cm) and herb layer, species name, height, cover, abundance, and other attributes were documented. In addition, environmental variables were recorded for each plot, including canopy closure, coordinates, elevation, slope, slope position, and slope aspect (Fang et al. 2009).

All plant species were identified and recorded in the field. For species that could not be identified on‐site, electronic specimens were collected, accompanied by close‐up photographs of key taxonomic features (including, at a minimum, flowers or fruits, leaf arrangement, and stem morphology), along with GPS coordinates. Species identification was conducted concerning the Flora Reipublicae Popularis Sinicae (http://www.iplant.cn/) and the Field Guide to Common Plants of China: Shandong Edition (Liu 2009). All recorded species were categorized into three major life‐form groups: trees, shrubs (including woody vines), and herbs (including herbaceous vines). Invasive species were identified based on the China Invasive Species Information System (http://www.iplant.cn/ias/protlist).

### 2.3 Environmental Factors

This study examined eight factors categorized into three groups: island attributes, landscape, and climate, as presented in Table 1. The island attributes included area (A), distance to the mainland (DM), and distance to the nearest island (DN). The data for A, DM, and DN were obtained from the vector map of the islands provided by the Shandong Changdao National Nature Reserve. In this context, DM and DN indicate the isolation of the islands.

The landscape is assessed using two key metrics: patch density (PD) and the human influence index (HII). PD quantifies the extent of landscape fragmentation per unit area and is calculated using the formula:

(1) PD=NP/A

In this formula, NP represents the total number of patches. HII measures the degree of human modification and utilization of the island, expressed as the ratio of the combined built‐up and cultivated areas to the island area. Land use data was sourced from the European Space Agency (ESA) WorldCover product (Zanaga et al. 2022), which provides detailed land use type information at a resolution of 10 m, ensuring accurate coverage of the islands examined.

Climate includes annual mean temperature (AMT), annual precipitation (AP), and annual mean wind (AMW). The AMT and AP data were sourced from the 1‐km resolution annual mean temperature dataset and the 1‐km resolution annual mean precipitation dataset for China, available on the Geographic Data Sharing Infrastructure, global resources data cloud (http://www.gis5g.com). The AMW data were obtained from the global 1‐km resolution historical wind speed dataset of WorldClim (Fick and Hijmans 2017).

All of the above environmental factors were extracted using the R package “sf” (Pebesma 2018; Pebesma and Bivand 2023).

### 2.4 Statistical Analysis

This study employs species richness, Shannon–Wiener diversity index (Shannon 1948), Simpson diversity index (Simpson 1949), and Pielou evenness index (Pielou 1966) to quantify the alpha diversity of vascular plants on the islands (Chen et al. 2024), represented by the formula:

(2) $$H=-\sum_{i=1}^{S}\left(P_{i}\ln P_{i}\right)$$

(3) $$D=1-\sum_{i=1}^{S}{P_{i}}^{2}$$

(4) J=H/lnS

(5) $$P_{i}=\frac{N_{i}}{N},$$

where *H* is the Shannon‐Wiener diversity index, *D* is the Simpson diversity index, *J* is the Pielou evenness index, *S* is the number of species, *P*_*i*_ is the relative abundance of the species *i*, *N* is the total number of individuals of all species, and *N*_*i*_ is the total number of individuals of the species *i*.

For beta diversity, we utilized Jaccard's dissimilarity index (Jaccard 1912), represented by the formula:

(6) $$C=1-a/\left(a+b+c\right),$$

where *a* is the number of species common to both islands, *b* is the number of species exclusive to the first island, and *c* is the number of species unique to the second island. A higher value of the Jaccard dissimilarity index indicates a greater difference in species composition between the islands. We further analyzed the variations in plant composition among the islands using non‐metric multidimensional scaling (NMDS) based on the Jaccard distance (Eibes et al. 2021) in the R package ‘vegan’ (Oksanen et al. 2024). The coefficient of Stress was employed to evaluate the fitting results of the NMDS analysis. When Stress < 0.2, the fitting result was fair; when Stress < 0.1, the fitting result was good (Clarke 1993; Dexter et al. 2018).

The covariance between environmental factors was assessed using the variance inflation factor (VIF) before model construction (Cheng et al. 2022). The VIF was calculated using the R package “usdm” (Naimi et al. 2014). It was found that there was significant covariance between DM and AMT. After excluding AMT from the analysis, we recalculated the VIF for the remaining seven environmental factors, all of which had VIF values below 10. This indicated that the covariance among the remaining factors was weak.

To investigate the effects of individual environmental factors on plant species richness, Shannon–Wiener diversity index, Simpson diversity index, and Pielou evenness index, general linear regression analyses were first performed. To improve the model's goodness of fit, log10 transformations were applied to plant species richness, A, DM, and DN (Shang et al. 2023). To further assess the combined effects of multiple environmental factors on plant alpha diversity, all environmental variables were standardized (mean = 0, standard deviation = 1) before modeling. This standardization was performed to eliminate the influence of differing measurement scales and to facilitate direct comparison of the relative effects of each variable. Subsequently, generalized linear models (GLMs) were constructed using the glm() function from the stats package in R (R Core Team 2024). Plant species richness was modeled using a Poisson distribution. Given their distributional characteristics, Shannon–Wiener diversity index, Pielou's evenness index, and Simpson diversity index were modeled using a Gamma distribution. In addition, the glmm.hp() function from the glmm.hp package in R was used to estimate the relative importance of each predictor variable in the multivariate models, thereby identifying the key environmental factors that predominantly influence the response of the different alpha diversity indices (Lai et al. 2023; Lai, Zou, Zhang, Zhang, and Mao 2022).

To investigate how environmental factors influence variation in species composition (i.e., beta diversity) across islands (Tuomisto and Ruokolainen 2006), detrended correspondence analysis (DCA) (Hill and Gauch 1980) was first performed using a species presence/absence data matrix (coded as 1 for presence and 0 for absence). Since the maximum gradient length was less than 3, redundancy analysis (RDA) was selected for subsequent analysis. RDA was conducted between the species presence/absence matrix and the environmental variable matrix (Yu et al. 2012; Yang et al. 2022). A Monte Carlo permutation test (999 permutations) was used to assess the statistical significance of the relationship between environmental factors and species distribution patterns across islands. To further evaluate the relative contribution of each environmental factor to species composition, the rdacca.hp. package in R was employed (Lai, Zou, Zhang, and Peres‐Neto 2022). Pearson's correlation coefficient was calculated to assess intercorrelations among environmental variables. In addition, the Mantel test is performed to explore the relationships between the environmental factors distance matrix, the geographical distance matrix, and the species composition distance matrix (i.e., beta diversity) to explain the variations in beta diversity (Fattorini 2010; Steinitz et al. 2006), which is a different object from that said by RDA (Tuomisto and Ruokolainen 2006). All RDA, Mantel tests, and visualizations were conducted using the R packages vegan (Oksanen et al. 2024), rdacca.hp. (Lai, Zou, Zhang, and Peres‐Neto 2022), ggcor (Huang et al. 2020), dplyr (Wickham et al. 2023), and ggplot2 (Wickham 2016).

## 3 Results

### 3.1 Species Composition and Distribution

A total of 485 vascular plant taxa, representing 88 families and 299 genera, were recorded from the 10 inhabited islands surveyed in this study (Table S2). This included 63 trees, 86 shrubs, 336 herbs, and 47 invasive plants.

At the family level, overall plant diversity was dominated by Asteraceae (69 taxa), Poaceae (53 taxa), and Fabaceae (40 taxa). Among trees, the most represented families were Fabaceae and Rosaceae (6 taxa each), followed by Sapindaceae and Ulmaceae (5 taxa each). In terms of shrubs, Fabaceae (17 taxa) and Rosaceae (14 taxa) were predominant. Herbs were chiefly represented by Asteraceae (69 taxa) and Poaceae (53 taxa). Invasive plants were mainly from Asteraceae (16 taxa) and Amaranthaceae (7 taxa).

At the genus level, the most species‐rich genera overall were *Artemisia* (10 taxa), *Lespedeza* (8 taxa), *Euphorbia* (7 taxa), and *Prunus* (7 taxa). For trees, *Quercus* and *Ulmus* were the most common genera (4 taxa each). In the shrub category, *Lespedeza* (8 taxa) and *Prunus* (5 taxa) were dominant, while herbs were largely represented by *Artemisia* (10 taxa) and *Euphorbia* (7 taxa). Invasive plants were mainly from *Amaranthus* (4 taxa) and *Erigeron* (3 taxa).

Plant species richness varied considerably across islands, ranging from 84 to 226 taxa. The number of tree species ranged from 10 to 33, shrubs from 13 to 49, herbs from 54 to 170, and invasive species from 4 to 25 (Table S3). Tuoji Island had the highest total plant and shrub richness. Nanchangshan Island hosted the most tree species and invasive species, while Daheishan Island had the highest herb species richness. In contrast, Xiaoheishan Island and Miaodao Island had the lowest total plant richness, Xiaoqin Island had the fewest tree species, Miaodao Island had the fewest shrub species, and Xiaoheishan Island had the fewest herbs. Both Xiaoheishan and Xiaoqin Islands had the lowest number of invasive species (Table S3).

Diversity index values varied substantially across islands and plant life forms (Table S4). For all vascular plants, the Shannon‐Wiener diversity index ranged from 1.40 on Nanchangshan Island to 3.13 on Daheishan Island. Daheishan Island also had the highest Simpson diversity index (0.92) and Pielou evenness index (0.73), indicating a highly diverse and balanced community. In contrast, Nanchangshan Island exhibited the lowest overall diversity and evenness (Simpson diversity index = 0.49, Pielou evenness index = 0.35).

Tree layer diversity was generally lower across islands. The highest Shannon‐Wiener diversity index for trees was observed on Daheishan Island (1.78), followed by Nanhuangcheng Island (1.28) and Miaodao Island (1.23). Xiaoqin Island had the lowest tree diversity, with a Shannon‐Wiener diversity index of 0.25 and a Simpson diversity index of only 0.09, suggesting dominance by a few species.

Shrub layer diversity was more evenly distributed. Miaodao and Nanhuangcheng Islands showed the highest Pielou evenness values (0.81 and 0.82, respectively), while Daheishan Island had the highest Shannon‐Wiener diversity index (2.33). Most islands showed consistently high Simpson values for shrubs (above 0.80), indicating structurally rich communities.

For herb layer, Daheishan Island again had the highest diversity (Shannon‐Wiener diversity index = 2.69; Simpson diversity index = 0.88), while Nanchangshan and Beichangshan Islands had the lowest values (Shannon‐Wiener diversity index = 1.12). Evenness values were also lowest on Nanchangshan Island (0.31), suggesting dominance by a few herb species. Overall, Daheishan and Tuoji Islands consistently exhibited high diversity indices across most life forms, while Nanchangshan and Xiaoqin Islands had notably lower diversity, particularly in tree and herb layers.

Jaccard's dissimilarity indices among the islands are presented in Table S5. Regarding the overall species composition of plants, the smallest Jaccard index (J‐index) was observed between Daqin Island and Beihuangcheng Island (0.534), while the largest was found between Daqin Island and Miaodao Island (0.782). For tree species composition, the smallest J‐index (0.333) occurred between Xiaoqin Island and Nanhuangcheng Island, and the largest (0.838) was between Xiaoqin Island and Nanchangshan Island. In terms of shrub species composition, the smallest J‐index (0.491) was recorded between the Tuoji Islands and Nanhuangcheng Island, whereas the largest (0.771) was between Daheishan Island and Xiaoheishan Island. For herbaceous species composition, the smallest J‐index (0.500) was between Daqin Island and Beihuangcheng Island, and the largest (0.820) was between Daqin Island and Miaodao Island. In terms of the species composition of invasive plants, the J index was lowest on Beihengcheng Island and Daqin Island (0.412) and highest on Xiaoheshan Island and Miaodao Island (0.923).

**FIGURE 2:**
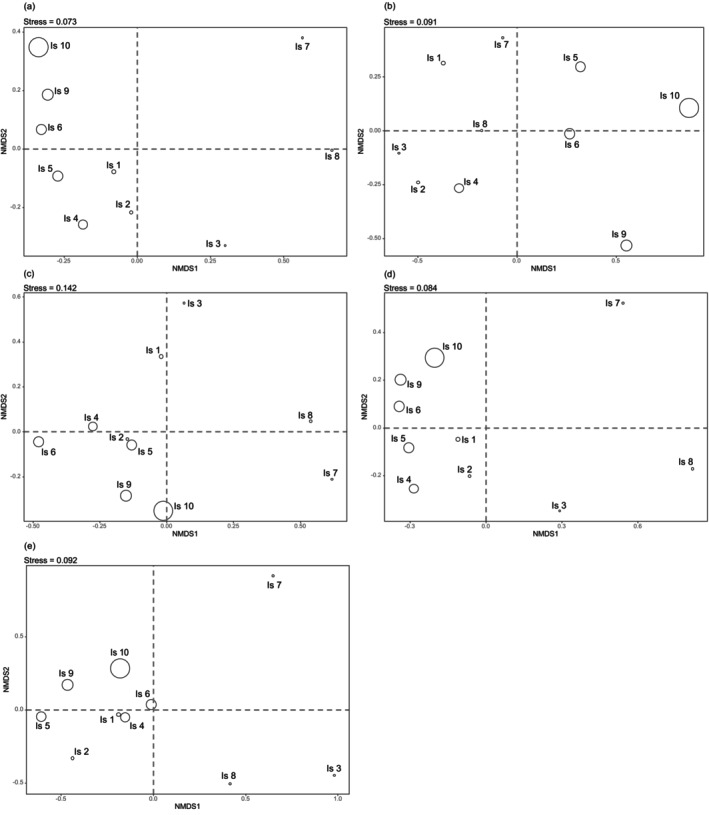
Non‐metric multidimensional scaling analysis assessed variations in plant composition among the islands. (a) All plants; (b) tree; (c) shrub; (d) herb; (e) invasive plants.

NMDS analysis results for all plants, each life form, and invasive plants are depicted in Figure 2. The stress values were 0.073, 0.091, 0.142, 0.084, and 0.092, respectively. These values indicate a good NMDS fit and reinforce the aforementioned results. The distribution of individual islands in the diagrams closely aligns with their actual geographic locations, showing that islands situated near one another generally exhibit a smaller J‐index between them.

### 3.2 Factors Affecting Plant Alpha Diversity

**FIGURE 3:**
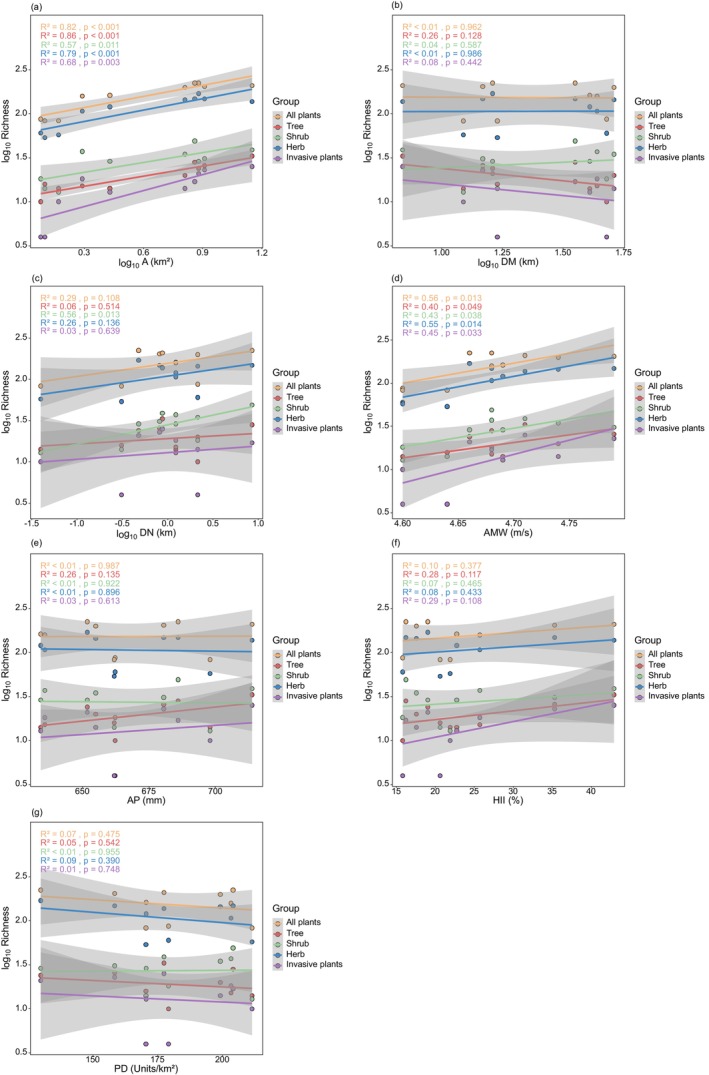
General linear regression analysis between plant richness and environmental factors. (a) Area (A); (b) distance to the mainland (DM); (c) distance to the nearest island (DN); (d) annual mean wind (AMW); (e) annual precipitation (AP); (f) human influence index (HII); (g) patch density (PD).

**FIGURE 4:**
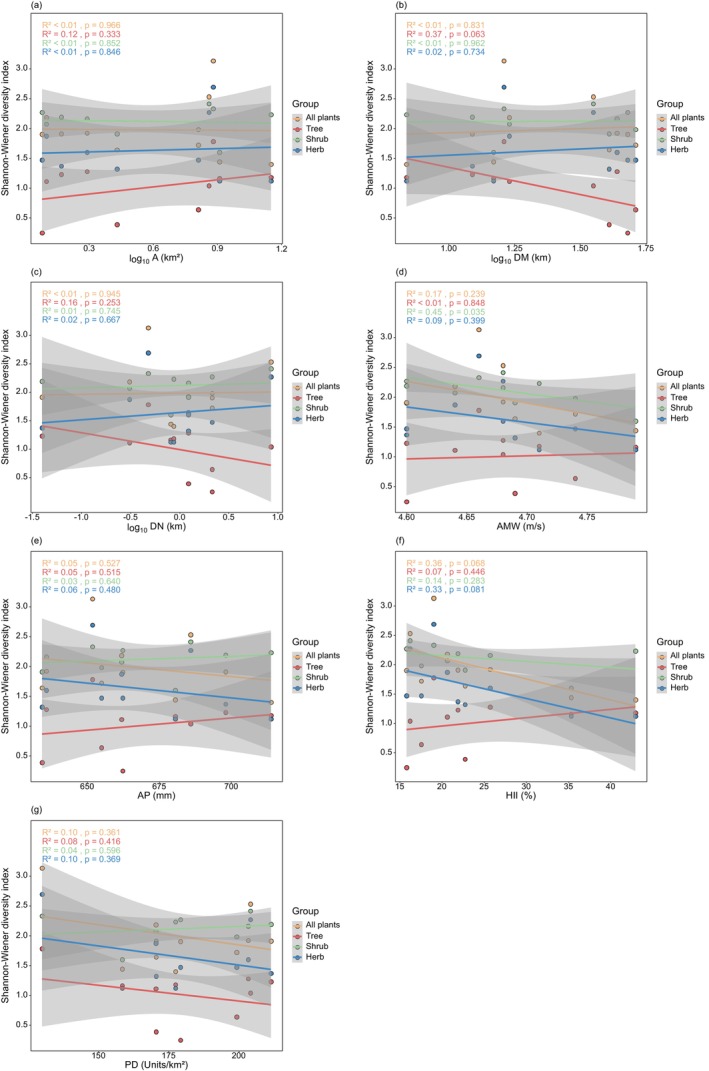
General linear regression analysis between Shannon‐Wiener diversity index and environmental factors. (a) Area (A); (b) distance to the mainland (DM); (c) distance to the nearest island (DN); (d) annual mean wind (AMW); (e) annual precipitation (AP); (f) human influence index (HII); (g) patch density (PD).

**FIGURE 5:**
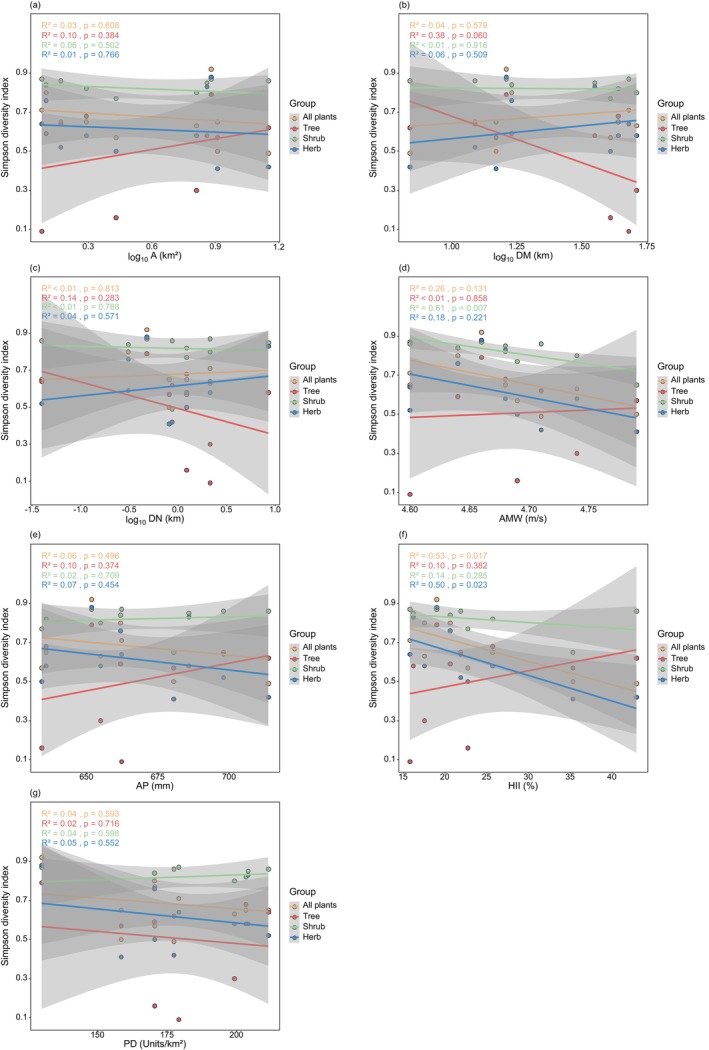
General linear regression analysis between Simpson diversity index and environmental factors. (a) Area (A); (b) distance to the mainland (DM); (c) distance to the nearest island (DN); (d) annual mean wind (AMW); (e) annual precipitation (AP); (f) human influence index (HII); (g) patch density (PD).

**FIGURE 6:**
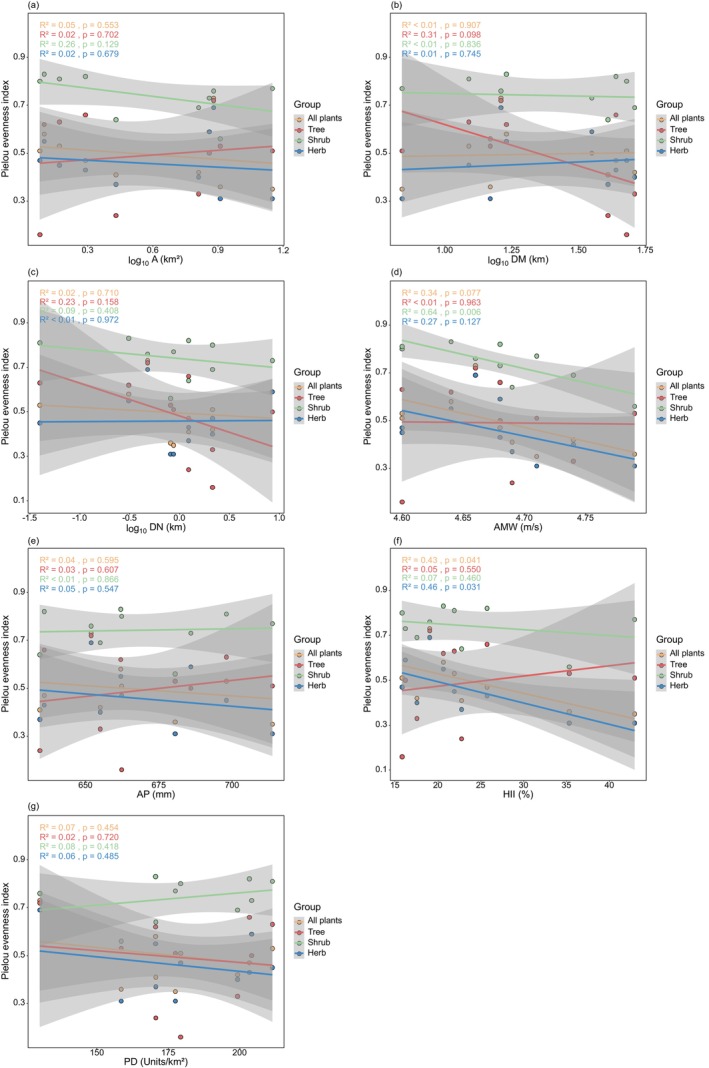
General linear regression analysis between Pielou evenness index and environmental factors. (a) Area (A); (b) distance to the mainland (DM); (c) distance to the nearest island (DN); (d) annual mean wind (AMW); (e) annual precipitation (AP); (f) human influence index (HII); (g) patch density (PD).

The results of the general linear regression analysis showed (Figure 3) that the richness of all plants, trees, shrubs, herbs, and invasive plants increased significantly with larger A and higher AMW, but was not significantly correlated with DM, AP, HII, and PD. Shrub richness significantly increased with greater DN, whereas all plants, trees, herbs, and invasive plants were not significantly correlated with DN. Overall, the richness of various plant species generally aligns with the response trends of environmental factors. In terms of diversity indices (Figures 4, 5, 6), the Shannon‐Wiener diversity index, Simpson diversity index, and Pielou evenness index for shrubs decreased significantly with increasing AMW; the Simpson diversity index and Pielou evenness index for all plants and herbs decreased significantly with increasing HII.

**FIGURE 7:**
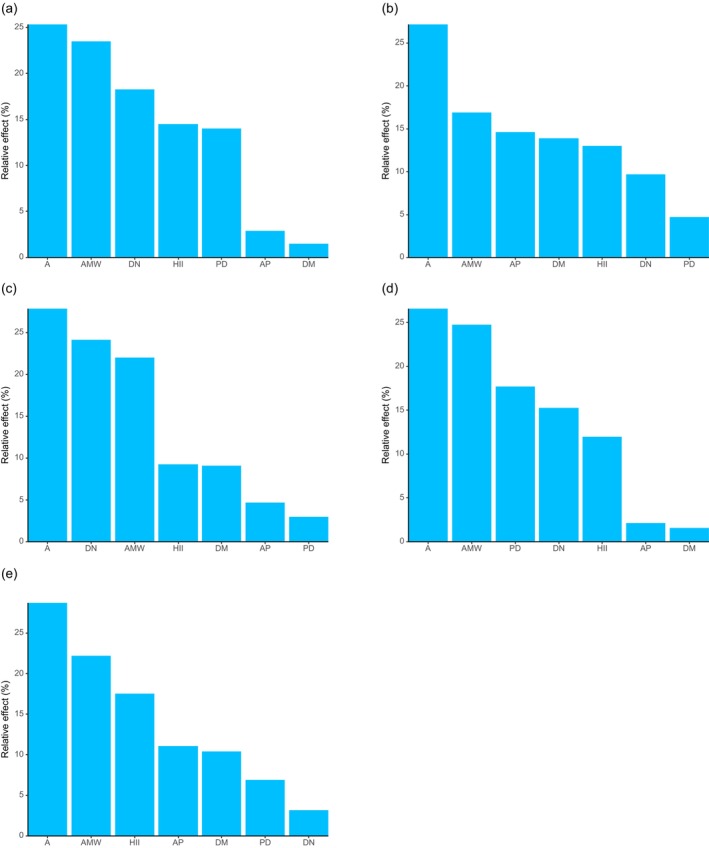
Relative importance of environmental variables in influencing plant richness. (a) All plants; (b) tree; (c) shrub; (d) herb; (e) invasive plants.

The results of the generalized linear model (Table S6) indicated that A had a significant positive effect on the richness of all plants, shrubs, herbs, and invasive species. DN also showed a significant positive correlation with the richness of all plants, shrubs, and herbs. AMW had a significant positive effect on the richness of all plants and herbs only. In contrast, AP exhibited a significant negative effect on the richness of all plants, shrubs, herbs, and invasive species. DM showed a significant negative effect solely on the richness of all plants. Notably, none of the environmental variables examined had a statistically significant effect on tree species richness. According to the variable importance ranking derived from the generalized linear mixed model (Figure 7), A consistently emerged as the most influential predictor for the richness of all plant life forms, including invasive species.

**FIGURE 8:**
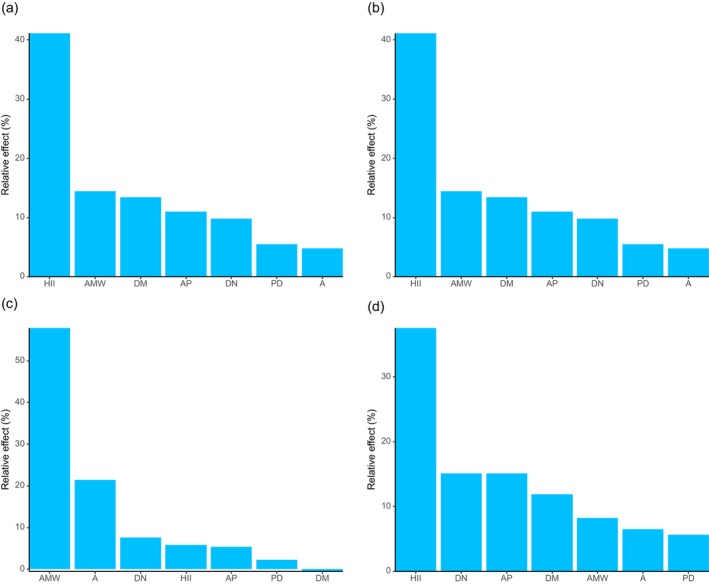
Relative importance of environmental variables in influencing Shannon‐Wiener diversity index. (a) All plants; (b) tree; (c) shrub; (d) herb.

**FIGURE 9:**
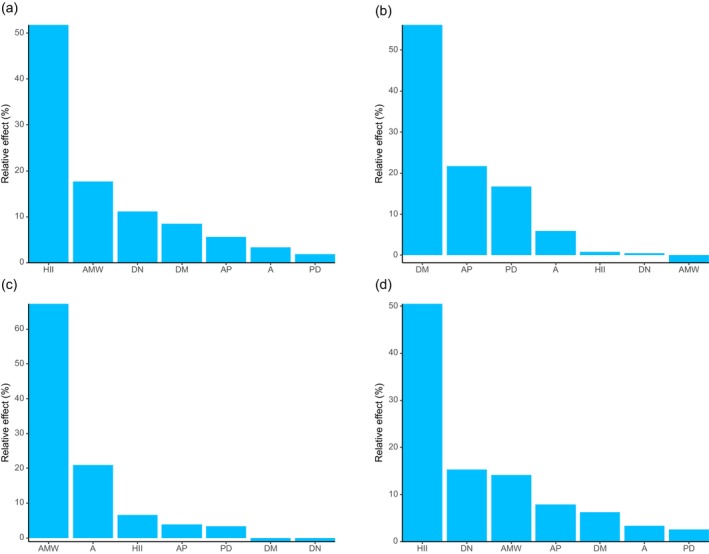
Relative importance of environmental variables in influencing Simpson diversity index. (a) All plants; (b) tree; (c) shrub; (d) herb.

**FIGURE 10:**
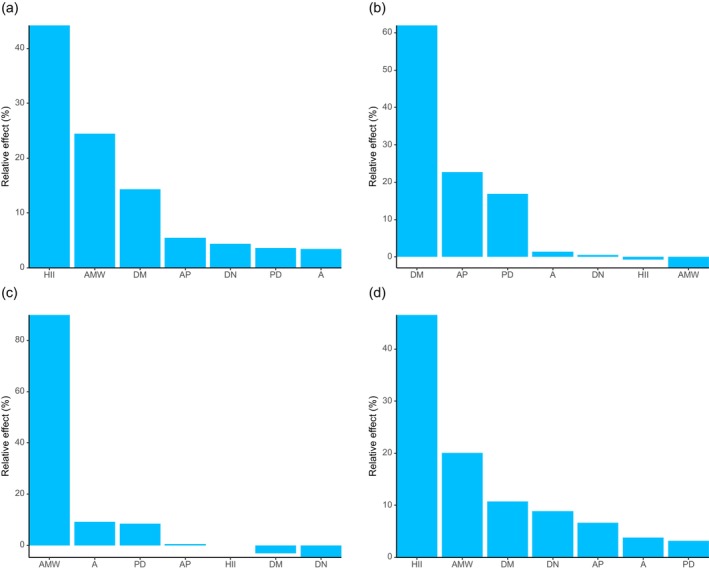
Relative importance of environmental variables in influencing Pielou evenness index. (a) All plants; (b) tree; (c) shrub; (d) herb.

Regarding diversity indices, the generalized linear model results (Tables S7–S9) showed that A had a significant positive effect on both the Shannon–Wiener and Simpson diversity indices for shrubs, while AMW had a significant negative effect on both indices. Further analysis using the glmm.hp function revealed that the most important predictor of the Shannon–Wiener diversity index varied across plant life forms (Figure 8): HII was the key factor for all plants, trees, and herbs, whereas AMW was the most influential predictor for shrubs. For the Simpson diversity index (Figure 9), HII was again the most important predictor for all plants and herbs, while DM played the dominant role for trees, and AMW was the most critical factor for shrubs. The key predictors for the Pielou evenness index (Figure 10) were consistent with those identified for the Simpson diversity index across the respective plant groups.

### 3.3 Factors Affecting Plant Beta Diversity

**FIGURE 11:**
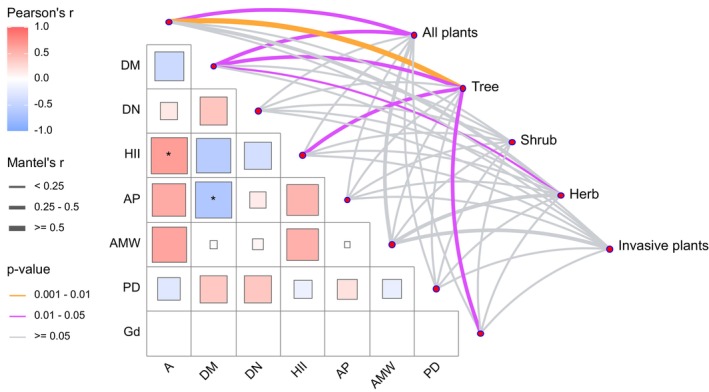
The Mantel test identified the factors impacting plant diversity.

The Mantel test results (Figure 11) revealed that A had a significant effect on the beta diversity of all plants and trees. DM significantly influenced the beta diversity of all plants, trees, and herbs. In contrast, HII and GD significantly affected only the beta diversity of trees. No environmental variable was found to have a significant effect on the beta diversity of shrubs or invasive species.

### 3.4 RDA Between Environmental Factors and Species Distribution

**FIGURE 12:**
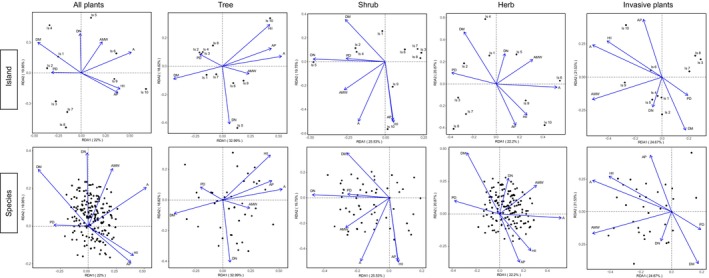
Redundancy analysis assessed the impact of environmental variables on plant distribution.

The RDA ordination diagram is shown in Figure 12; the cumulative explanatory power of the first two axes is 41.95%, 51.81%, 45.28%, 43.07%, and 46.20%, respectively. Vascular plant species are mainly distributed across quadrants 1, 2, and 4, with relatively few species appearing in quadrant 3. Islands with lower species richness (*N*) tend to cluster in quadrant 3, whereas islands with higher *N* values are predominantly located in quadrants 1 and 4. This pattern suggests that higher A, HII, and AP are generally associated with greater species richness.

Tree species are primarily distributed in quadrants 1 and 4, with islands exhibiting high *N* values concentrated in quadrant 4. This implies that high values of A, AP, HII, and AMW are positively related to tree species richness. Shrub species are mainly located in quadrants 2 and 3; however, islands with higher *N* values for shrubs are also concentrated in quadrant 4, indicating a positive association between shrub richness and factors such as A, AP, and HII.

Herbaceous plants are largely found in quadrants 1 and 4, suggesting that islands with high A and AMW values tend to support greater herbaceous species richness. In contrast, invasive species are mainly distributed in quadrants 2 and 3, and the islands with high invasive plant richness are likewise concentrated in these quadrants. This indicates that A, AMW, and HII are important predictors of invasive plant richness on the islands.

According to Table S10, A had a significant effect on the species distribution of all plant types, including trees, shrubs, herbs, and invasive species. DM significantly influenced the distribution of all plant types except invasive species. Additionally, HII significantly affected the species distribution of trees, shrubs, and invasive plants; AP significantly influenced trees and shrubs; and AMW significantly influenced only the distribution of invasive species. Other environmental variables did not show significant effects on species distribution.

**FIGURE 13:**
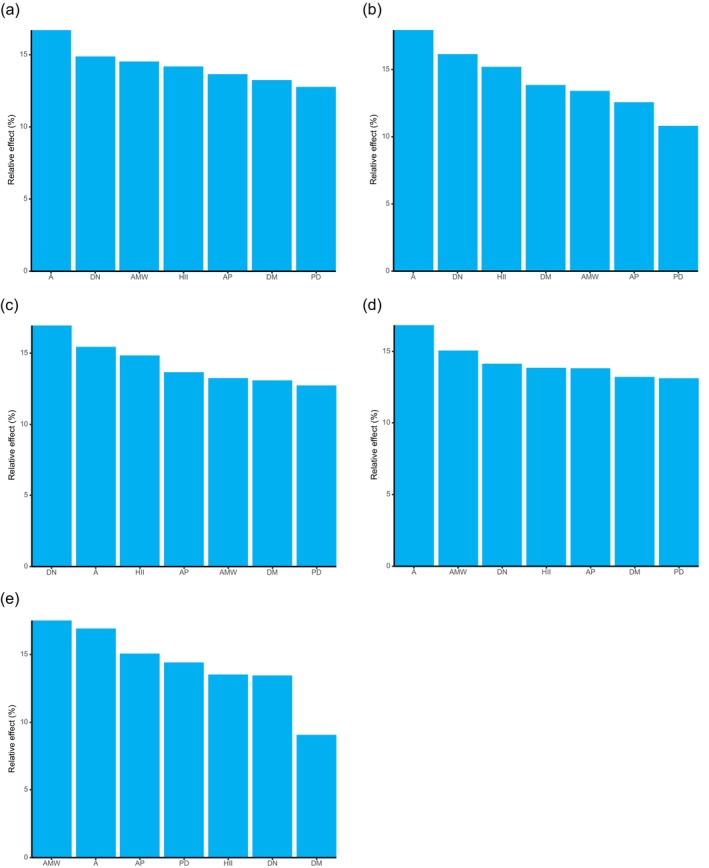
Relative importance of environmental variables in influencing the plant distribution. (a) All plants; (b) tree; (c) shrub; (d) herb; (e) invasive plants.

As shown in Figure 13, the relative importance of different environmental variables in shaping species distribution is ranked as follows:

For all plant species: A > DN > AMW > HII > AP > DM >PD.

For trees: A > DN >HII > DM> AMW > AP > PD.

For shrubs: DN > A > HII > AP > AMW > DM > PD.

For herbs: A > AMW > DN > HII > AP > DM > PD.

For invasive plants: AMW > A > AP > PD > HII > DN > DM.

Overall, A emerged as the most important predictive factor influencing the species distribution across different plant life forms.

## 4 Discussion

### 4.1 Effects of Environmental Factors on Vascular Plant Alpha Diversity on Islands

In this study, we found that the species richness of all plant types increased significantly with island area. This result is consistent with the relationship between area and species richness in island biogeography theory (Macarthur and Wilson 2001), suggesting that this principle holds even in the Miaodao Archipelago, despite substantial human disturbance. This result is also supported in other inhabited island studies. For instance, a study of 35 islands in the Sanyang Wetland of Wenzhou, Zhejiang, revealed that the species richness of varied life forms increased with island area (Liu et al. 2023). In the Zhoushan Archipelago, an increase in the area of 37 islands directly contributed to greater plant richness (Xu et al. 2023). Similarly, plant species richness in the Shengsi Islands was found to be directly proportional to the area of the islands (Ma et al. 2023). Overall, when considering various environmental factors, island area remains the most important predictor of plant species richness. This result has been confirmed in other studies of similar islands, such as the Ionian Islands (Valli et al. 2019), Thousand Island Lake (Yu et al. 2012), and Central Mediterranean islands (Testolin et al. 2024).

The analysis of species richness and isolation revealed that neither distance to the mainland (DM) nor distance to the nearest island (DN) was strongly correlated with species richness on the inhabited islands of the Miaodao Archipelago. Only DN exhibited a significant positive relationship with shrub species richness, which runs counter to the predictions of classical island biogeography theory (Macarthur and Wilson 2001). This may reflect long‐distance seed dispersal, whereby species with strong dispersal abilities colonize nearby islands non‐sequentially (Cain et al. 2000; Viana et al. 2016; Nogales et al. 2012). DN significantly affects plant richness only when other environmental factors are considered together, and the relative contribution of DN is low compared to A and AMW (Liu et al. 2023). Thus, we hypothesize that DM and DN do not accurately reflect the isolation of islands in the Miaodao Archipelago, where human activity is prevalent. Instead, we believe that the level of isolation may be more influenced by the intensity of human activities. Stronger human activities could facilitate the spread of certain plants, thereby diminishing the effect of isolation on plant richness (Ma et al. 2023). In addition, this may be due to the relatively short distances of DM and DN (the largest DM and DN were 50.92 and 8.48 km, respectively), which are not enough to restrict the spread of most vascular plants. This phenomenon also exists in other similar islands; research on the habitat islands of Thousand Island Lake revealed that isolation did not have a significant impact on the richness of plants (Hu et al. 2011). Similarly, studies on the small coastal islands of Wenzhou found no significant relationship between plant richness and distance from the mainland (Wang et al. 2017). Additionally, distance from the mainland was not a significant driver of plant richness in the Shengsi Archipelago (Ma et al. 2023).

Although DM showed no significant correlation with species richness, it was identified as the most important predictor of tree community structure, particularly affecting the Simpson diversity index and Pielou evenness index. This suggests that while distance from the mainland may not directly limit the total number of tree species, it plays a substantial role in shaping community‐level structural balance. Increased geographic isolation may hinder the natural dispersal and recruitment of certain tree species (Warren et al. 2015), enabling stress‐tolerant or anthropogenically introduced species to dominate, which results in reduced evenness and diversity within the community (Lambdon et al. 2008; Rojas‐Sandoval et al. 2022).

In addition to area and isolation, climatic factors were also important factors influencing plant alpha diversity in this study. We found that the species richness of all plants and different life forms had a significant positive correlation with AMW, and AMW significantly influenced plant richness, second only to A in relative contribution. However, AMW contributed more to shrubs, herbs, and invasive plant species richness than to trees, possibly because the habitat conditions of islands are dominated by strong winds, high sunshine, high evaporation, etc. Shrubs, herbs, and invasive plants are better adapted to strong winds than trees due to their short height and wind‐dispersed seeds, and they are more conducive to living on islands with high wind speeds (Tamme et al. 2014; Thomson et al. 2011). However, the Shannon‐Wiener diversity index, Simpson diversity index, and Pielou evenness index for shrubs all significantly declined with increasing AMW, and AMW was identified as the most important predictor. This indicates that wind exerts complex and multidimensional ecological effects on shrub community structure. The phenomenon may be driven by wind‐imposed selective pressure on species composition: in high‐wind environments, species with strong wind resistance are more likely to dominate ecological niches, while less tolerant species are excluded (Papaik and Canham 2006). While species richness may increase due to environmental filtering favoring a subset of colonizers, diversity and evenness often decline, reflecting a shift from a “diverse and balanced” community to one with more species but dominated by a few (Hillebrand et al. 2008). Thus, AMW has a dual ecological role in shaping shrub communities: it can facilitate species colonization while simultaneously increasing dominance inequality.

Although AP had a significant effect on plant richness when all environmental factors were integrated, its relative contribution was low, which resulted in a non‐significant linear correlation between plant richness and AP. Additionally, there were differences in the response of different life forms to AP, as reflected in the fact that AP contributed more to trees than to shrubs, herbs, and invasive plants. The tree richness tended to increase with AP, which may be related to the fact that shrubs, herbs, and invasive plants have wider ecological niches, while trees need to survive in environments where resources are more stable, and trees are more sensitive to AP (Šímová et al. 2018; Pierce et al. 2017).

In the Miaodao Islands, HII, PD, and plant species richness were not significantly correlated, and their relative contribution rates were low. This finding is consistent with previous studies, which have also pointed out that in contexts where human activity is frequent or ecosystems have already been significantly disturbed by humans, species richness may respond slowly or insignificantly to human disturbance (Shang et al. 2023; Xie et al. 2023). However, further analysis indicates that the impact of HII on community structure remains significant. Specifically, we found that the Simpson diversity index and Pielou evenness index for all plants and the herbaceous layer decreased significantly with increasing HII, and HII was identified as the most important predictor in the relevant models. This suggests that although human disturbance did not significantly affect species numbers themselves, it significantly disrupted the balance and dominance patterns of the internal structure of the community (Chaneton and Facelli 1991). In areas with high human activity intensity, vegetation structure is often subjected to multiple disturbances, including habitat fragmentation (De Lima Filho et al. 2021; Scanes 2018; Mullu 2016), reduced resource availability, and the introduction of non‐native species (Vitousek et al. 1997; Zimmermann et al. 2014). These disturbances lead to the expansion of dominant species that are highly adaptive or competitively advantaged, while sensitive rare species decline or even become locally extinct. As a result, communities tend to become structurally simpler, characterized by reduced species distribution uniformity, increased dominance, and ultimately reflected in decreased Simpson diversity indices and Pielou evenness indices. Research conducted on the Pingtan Islands has also found that human activities negatively impact island effects and habitat heterogeneity on diversity (Chen et al. 2024). This underscores the urgent need for heightened attention to this issue in ecological conservation and management efforts (Newbold et al. 2015).

Therefore, considering the effects on diversity indicators such as species complexity and species evenness can help us understand more comprehensively the changing patterns of island plant diversity in the context of the human world (Helmus et al. 2014).

### 4.2 Effects of Environmental Factors on the Distribution of Plant Species on Islands

The results of the RDA indicate that the interactive effects of multiple environmental factors significantly influence the distribution of island plants. Among these factors, A has the strongest influence on the distribution of all plants, trees, shrubs, herbs, and invasive plants. As the area of an island increases, it tends to enhance the spatial heterogeneity of sunlight, water, and soil availability. This variation creates diverse microhabitats that are suitable for different species, thus contributing to the variability in plant distributions (Lu et al. 2011).

Additionally, AMW plays a crucial role in shaping the spatial distribution patterns of invasive plants. Invasive plants often possess strong dispersal capabilities, with wind‐mediated dispersal being a primary mechanism of dispersal (Zhang et al. 2023). Higher wind speeds not only facilitate the long‐distance dispersal of seeds but may also enhance their chances of establishing and expanding in new environments (Pazos et al. 2013; Soons et al. 2004). These findings suggest that, in addressing invasive plant management and control, it is important to focus on the impact of wind factors on their dispersal pathways and dispersal risks.

HII and AP also had significant effects on the distributions of trees, shrubs, and invasive plants, with moderate relative contributions. Most of the forests on islands are plantations, and the pre‐construction of plantations tends to give more consideration to trees and shrubs, which leads to a high influence of anthropogenic disturbance on the species selection of trees and shrubs (Chi et al. 2016, 2018). At the same time, human activities (such as infrastructure construction, tourist activities, and material transportation) may also inadvertently introduce alien plants into island ecosystems, thereby promoting their colonization and spread (Reaser et al. 2007; Delgado et al. 2017; Dimitrakopoulos et al. 2022).

Additionally, AP typically reflects the level of moisture or water resource abundance in a region. The ecological niches of trees and shrubs tend to be narrower and more sensitive to moisture levels, which further affects the distribution of trees and shrubs (Šímová et al. 2018). DM had a significant effect on the distribution of all plants, trees, shrubs, and herbs, but its relative contribution was low, suggesting that it was a secondary driver. We hypothesize that DM has a limiting effect on the dispersal of a few plant species, thus affecting species distribution. However, the number of species affected by DM is small, which makes it a minor driver compared to other factors. Therefore, the protection of larger islands should be enhanced in the Miaodao Archipelago to conserve more plant species by maintaining diverse habitats and reducing human disturbance (Shang et al. 2023).

### 4.3 Effects of Environmental Factors on the Beta Diversity of Island Plants

The ecological niche differentiation indicates that variations in species composition between two regions are positively correlated with the environmental differences between them. In other words, greater habitat heterogeneity leads to higher β diversity (Xing and He 2019; Tuomisto et al. 2003). The habitat diversity hypothesis posits that larger islands contain more habitats, which in turn provide a greater number of ecological niches compared to smaller islands (Chen et al. 2020; Liu et al. 2018). Consequently, when the area difference between two islands is smaller, their environmental differences are also smaller, leading to more similar species compositions. In this study, A was found to have a significant correlation with the beta diversity of all plants and trees. This observation aligns with ecological niche theory and has been supported by other studies conducted on islands (Valli et al. 2019; Liu et al. 2020; Hu et al. 2019). The common shrub species in the Miaodao Archipelago predominantly belong to the Fabaceae and Rosaceae, while the prevalent herb species are mainly from the Asteraceae and Poaceae. We hypothesize that the lack of significant correlation between shrub, herb, and invasive plant's beta diversity and A is likely due to their strong environmental adaptability, which is not limited by the island area. Human impacts also play a role in the ecological niche process; in our study, HII was significantly correlated with the beta diversity of trees. Land use has led to significant habitat fragmentation on the islands, resulting in the local extinction of small populations of native species (Newbold et al. 2015). Furthermore, the introduction of numerous new species, such as plantation forest species and garden species, has altered the island's species pool (Van Kleunen et al. 2015). These cases suggest that trees are relatively more vulnerable to human activities.

The dispersal process suggests that a species' distribution is primarily determined by its ability to disperse, making distance the main limiting factor in the formation of species beta diversity. GD is significantly related to the beta diversity of trees. Animals are the primary dispersers of tree seeds, while bird species often disperse seeds on islands. As the distance between islands increases, the extent and direction of animal dispersal become limited, resulting in a decreased rate of species exchange between islands. Consequently, the differences in species composition among islands tend to be greater. This finding aligns with results from other studies (Cabral et al. 2014; Chi, Sun, et al. 2019; Kubota et al. 2014). Additionally, DM is significantly correlated with the β diversity of all plants, trees, and herbs. DM is somewhat representative of the degree of isolation of an island, so when the difference in the distance from the mainland between two islands is small, the rates of species immigration and emigration become similar, leading to more similar species compositions on those islands. This phenomenon has also been confirmed in other studies (Ibanez et al. 2018; Liu et al. 2019). In summary, all plants and trees are driven by both ecological niche differentiation and dispersal processes, while herbs are mainly limited by dispersal.

## 5 Conclusion

Based on field survey data from 10 representative inhabited islands in the Miaodao Archipelago, this study systematically evaluated the effects of natural and anthropogenic factors on island plant diversity from the perspectives of alpha diversity, beta diversity, and species distribution. The results demonstrated that island area was the most critical determinant of species richness across plant life forms, confirming the applicability of island biogeography theory even under substantial human disturbance. Annual mean wind speed significantly and positively influenced the richness of herbs, shrubs, and invasive species but negatively affected the structural evenness of shrub communities. Although the human influence index did not significantly impact species richness, it showed strong negative effects on Simpson diversity and Pielou evenness indices, indicating notable disruptions to community structural balance.

Beta diversity analyses revealed that ecological niche differentiation and dispersal limitation jointly structured the turnover patterns of all plant species and trees. In contrast, herbaceous beta diversity was predominantly shaped by dispersal processes. Island area, human disturbance, and inter‐island geographic distance significantly influenced species turnover, with tree communities being particularly sensitive to habitat heterogeneity and spatial isolation.

Redundancy analysis further showed that the island area exhibited the highest explanatory power across all plant groups. Wind speed was the primary driver of invasive plant distribution, while human disturbance and precipitation mainly influenced the spatial distribution of trees and shrubs. These results highlight distinct environmental responses among different plant life forms.

Overall, the spatial patterns of plant diversity in the Miaodao Archipelago are jointly shaped by multidimensional environmental factors. The coupled effects of natural attributes and anthropogenic disturbance play a central role in community assembly processes across life forms. This study underscores the necessity of prioritizing the conservation of larger islands, maintaining habitat heterogeneity, and strengthening the management of human activities and invasive species to safeguard biodiversity and sustain ecological stability in temperate island ecosystems.

## Author Contributions

**Haitao Yu:** conceptualization (equal), data curation (lead), formal analysis (lead), investigation (equal), methodology (lead), software (lead), validation (lead), visualization (lead), writing – original draft (lead). **Xue Feng:** investigation (equal), visualization (supporting). **Yuhuang Lin:** investigation (equal), visualization (supporting). **Ying Yang:** investigation (equal). **Zixiong Song:** investigation (equal). **Shie Ching Ang:** investigation (equal). **Qingchun Wang:** conceptualization (equal), funding acquisition (lead), methodology (supporting), supervision (lead), writing – review and editing (lead).

## Conflicts of Interest

The authors declare no conflicts of interest.

## Supporting information


**Tables S1–S10:** ece372329‐sup‐0001‐AppendixS1.docx.

## Data Availability

The data have been submitted to figshare, and the link is https://doi.org/10.6084/m9.figshare.28749668.
