# Supplementary material for: Island Biogeography in the Anthropocene: Natural and Anthropogenic Drivers of Plant Diversity in the Miaodao Archipelago
Source: Ecol Evol. 2025 Oct 17;15(10):e72329. doi: 10.1002/ece3.72329 (PMC12531715; doi:10.1002/ece3.72329)
Supplement: Supplementary file 1 — Tables S1–S10: ece372329‐sup‐0001‐AppendixS1.docx. [file ECE3-15-e72329-s001.docx]

**Island biogeography in the Anthropocene: natural and anthropogenic drivers of plant diversity in the Miaodao Archipelago**

Haitao Yu ^a^, Xue Feng ^a^, Yuhuang Lin ^a^, Ying Yang ^a^, Zixiong Song ^a^, Shie Ching Ang ^a^, Qingchun Wang ^a,*^

^a^ School of Ecology and Nature Conservation, Beijing Forestry University, Beijing, 100083, China

*Corresponding author.

E-mail address: wangqch96@aliyun.com (Qingchun Wang), yht010422@gamil.com (Haitao Yu)

**Table S1**. Sample site characteristics statistics table of the inhabited islands of the Miaodao Archipelago.

| **Sample** | **Island** | **E(°)** | **N(°)** | **Elevation(m)** | **Slope(°)** | **Slope position** | **Dominant species** |
| --- | --- | --- | --- | --- | --- | --- | --- |
| 1 | Beihuangcheng Island | 120.92173220 | 38.38767769 | 64.0 | 24.0 | Up | *Pinus thunbergii* |
| 2 | Beihuangcheng Island | 120.91099469 | 38.39723416 | 147.4 | 14.0 | Up | *Pinus thunbergii* |
| 3 | Beihuangcheng Island | 120.91516987 | 38.39070157 | 103.0 | 29.3 | Up | *Pinus thunbergii* |
| 4 | Beihuangcheng Island | 120.89852309 | 38.38552064 | 53.1 | 23.4 | Mid | *Pinus thunbergii* |
| 5 | Beichangshan Island | 120.71818344 | 37.98138881 | 78.1 | 22.0 | Up | *Pinus thunbergii* |
| 6 | Beichangshan Island | 120.69276180 | 37.99112253 | 76.9 | 22.0 | Up | *Pinus thunbergii* |
| 7 | Beichangshan Island | 120.72305922 | 37.97072788 | 44.2 | 15.3 | Down | *Platycladus orientalis* |
| 8 | Beichangshan Island | 120.70244933 | 37.98064107 | 113.6 | 25.2 | Up | *Pinus thunbergii* |
| 9 | Beichangshan Island | 120.70485528 | 37.97363029 | 171.6 | 28.0 | Up | *Quercus acutissima* |
| 10 | Daheishan Island | 120.60291739 | 37.96802753 | 91.2 | 15.0 | Up | *Robinia pseudoacacia* |
| 11 | Daheishan Island | 120.60842499 | 37.96736812 | 101.8 | 15.4 | Up | *Pinus thunbergii* |
| 12 | Daheishan Island | 120.61492693 | 37.97573335 | 90.9 | 18.0 | Up | *Pinus thunbergii* |
| 13 | Daheishan Island | 120.61598823 | 37.96298672 | 60.5 | 27.6 | Up | *Quercus acutissima* |
| 14 | Daheishan Island | 120.61657292 | 37.95593429 | 28.5 | 1.8 | Down | *Paulownia tomentosa* |
| 15 | Daheishan Island | 120.62061485 | 37.96811426 | 47.6 | 13.0 | Up | *Platycladus orientalis* |
| 16 | Daqin Island | 120.81575601 | 38.30388045 | 42.8 | 13.0 | Down | *Robinia pseudoacacia* |
| 17 | Daqin Island | 120.83175279 | 38.31306751 | 115.1 | 26.0 | Up | *Pinus thunbergii* |
| 18 | Daqin Island | 120.80324601 | 38.29475351 | 58.8 | 22.9 | Up | *Robinia pseudoacacia* |
| 19 | Daqin Island | 120.81491623 | 38.31193896 | 67.2 | 30.8 | Down | *Robinia pseudoacacia* |
| 20 | Miaodao Island | 120.68310255 | 37.94427793 | 45.6 | 26.9 | Mid | *Pinus thunbergii* |
| 21 | Miaodao Island | 120.67979133 | 37.93687788 | 73.0 | 20.8 | Up | *Quercus acutissima* |
| 22 | Nanhuangcheng Island | 120.91240546 | 38.34630872 | 44.5 | 14.0 | Up | *Quercus acutissima* |
| 23 | Nanhuangcheng Island | 120.90304160 | 38.35283353 | 45.9 | 15.0 | Up | *Pinus thunbergii* |
| 24 | Nanhuangcheng Island | 120.89893800 | 38.35995123 | 32.2 | 20.0 | Down | *Robinia pseudoacacia* |
| 25 | Nanhuangcheng Island | 120.90328717 | 38.36625465 | 86.4 | 24.0 | Up | *Pinus thunbergii* |
| 26 | Nanchangshan Island | 120.74730213 | 37.93180889 | 93.8 | 14.0 | Up | *Pinus thunbergii* |
| 27 | Nanchangshan Island | 120.75053636 | 37.91864849 | 83.3 | 23.4 | Mid | *Quercus acutissima* |
| 28 | Nanchangshan Island | 120.75325386 | 37.90487372 | 61.5 | 21.0 | Up | *Robinia pseudoacacia* |
| 29 | Nanchangshan Island | 120.75503648 | 37.89852386 | 73.0 | 27.9 | Up | *Pinus thunbergii* |
| 30 | Nanchangshan Island | 120.73692227 | 37.93983527 | 92.4 | 28.1 | Up | *Pinus thunbergii* |
| 31 | Tuoji Island | 120.76950089 | 38.16201368 | 76.2 | 34.7 | Up | *Pinus thunbergii* |
| 32 | Tuoji Island | 120.76702732 | 38.16777694 | 62.4 | 19.3 | Up | *Pinus thunbergii* |
| 33 | Tuoji Island | 120.73946659 | 38.17520238 | 89.8 | 0.0 | Mid | *Robinia pseudoacacia* |
| 34 | Tuoji Island | 120.74433578 | 38.16857967 | 68.9 | 28.9 | Up | *Robinia pseudoacacia* |
| 35 | Tuoji Island | 120.74034294 | 38.18109779 | 116.9 | 25.4 | Up | *Robinia pseudoacacia* |
| 36 | Tuoji Island | 120.73864203 | 38.16151531 | 98.1 | 29.6 | Up | *Pinus thunbergii* |
| 37 | Xiaoheishan Island | 120.64998139 | 37.97199027 | 79.3 | 14.0 | Up | *Pinus thunbergii* |
| 38 | Xiaoheishan Island | 120.64756734 | 37.97619949 | 55.1 | 20.2 | Up | *Robinia pseudoacacia* |
| 39 | Xiaoqin Island | 120.84128083 | 38.34858139 | 99.0 | 11.0 | Up | *Pinus thunbergii* |
| 40 | Xiaoqin Island | 120.84371562 | 38.34474441 | 70.8 | 25.8 | Mid | *Pinus thunbergii* |

**Table S2. The checklist of vascular plants of the inhabited islands of the Miaodao Archipelago.**

| **Family** | **Genus** | **Species** |
| --- | --- | --- |
| Actinidiaceae | *Actinidia* | *Actinidia chinensis* |
| Amaranthaceae | *Achyranthes* | *Achyranthes bidentata* |
| Amaranthaceae | *Alternanthera* | *Alternanthera philoxeroides* |
| Amaranthaceae | *Amaranthus* | *Amaranthus blitum* |
| Amaranthaceae | *Amaranthus* | *Amaranthus cruentus* |
| Amaranthaceae | *Amaranthus* | *Amaranthus hybridus* |
| Amaranthaceae | *Amaranthus* | *Amaranthus viridis* |
| Amaranthaceae | *Bassia* | *Bassia scoparia* |
| Amaranthaceae | *Chenopodiastrum* | *Chenopodiastrum hybridum* |
| Amaranthaceae | *Chenopodium* | *Chenopodium album* |
| Amaranthaceae | *Chenopodium* | *Chenopodium ficifolium* |
| Amaranthaceae | *Corispermum* | *Corispermum puberulum* |
| Amaranthaceae | *Kali* | *Kali collinum* |
| Amaranthaceae | *Oxybasis* | *Oxybasis glauca* |
| Amaranthaceae | *Suaeda* | *Suaeda glauca* |
| Amaranthaceae | *Suaeda* | *Suaeda salsa* |
| Amaryllidaceae | *Allium* | *Allium fistulosum* |
| Amaryllidaceae | *Allium* | *Allium macrostemon* |
| Amaryllidaceae | *Allium* | *Allium neriniflorum* |
| Amaryllidaceae | *Allium* | *Allium ramosum* |
| Amaryllidaceae | *Allium* | *Allium tenuissimum* |
| Anacardiaceae | *Cotinus* | *Cotinus coggygria* var. *cinereus* |
| Anacardiaceae | *Rhus* | *Rhus chinensis* |
| Anacardiaceae | *Rhus* | *Rhus typhina* |
| Apiaceae | *Coriandrum* | *Coriandrum sativum* |
| Apiaceae | *Daucus* | *Daucus carota* |
| Apiaceae | *Foeniculum* | *Foeniculum vulgare* |
| Apiaceae | *Saposhnikovia* | *Saposhnikovia divaricata* |
| Apocynaceae | *Apocynum* | *Apocynum venetum* |
| Apocynaceae | *Cynanchum* | *Cynanchum auriculatum* |
| Apocynaceae | *Cynanchum* | *Cynanchum chinense* |
| Apocynaceae | *Cynanchum* | *Cynanchum rostellatum* |
| Apocynaceae | *Cynanchum* | *Cynanchum thesioides* |
| Apocynaceae | *Cynanchum* | *Cynanchum wilfordii* |
| Apocynaceae | *Periploca* | *Periploca sepium* |
| Apocynaceae | *Vincetoxicum* | *Vincetoxicum pycnostelma* |
| Apocynaceae | *Vincetoxicum* | *Vincetoxicum versicolor* |
| Araceae | *Pinellia* | *Pinellia ternata* |
| Araliaceae | *Hedera* | *Hedera nepalensis* var. *sinensis* |
| Aristolochiaceae | *Aristolochia* | *Aristolochia contorta* |
| Asparagaceae | *Anemarrhena* | *Anemarrhena asphodeloides* |
| Asparagaceae | *Asparagus* | *Asparagus brachyphyllus* |
| Asparagaceae | *Asparagus* | *Asparagus dauricus* |
| Asparagaceae | *Asparagus* | *Asparagus longiflorus* |
| Asparagaceae | *Asparagus* | *Asparagus oligoclonos* |
| Asparagaceae | *Barnardia* | *Barnardia japonica* |
| Asparagaceae | *Liriope* | *Liriope graminifolia* |
| Asparagaceae | *Liriope* | *Liriope spicata* |
| Asparagaceae | *Ophiopogon* | *Ophiopogon japonicus* |
| Asparagaceae | *Polygonatum* | *Polygonatum macropodum* |
| Asparagaceae | *Polygonatum* | *Polygonatum odoratum* |
| Asparagaceae | *Polygonatum* | *Polygonatum sibiricum* |
| Asparagaceae | *Yucca* | *Yucca gloriosa* |
| Asphodelaceae | *Hemerocallis* | *Hemerocallis citrina* |
| Asphodelaceae | *Hemerocallis* | *Hemerocallis minor* |
| Asteraceae | *Ageratum* | *Ageratum conyzoides* |
| Asteraceae | *Artemisia* | *Artemisia annua* |
| Asteraceae | *Artemisia* | *Artemisia argyi* |
| Asteraceae | *Artemisia* | *Artemisia capillaris* |
| Asteraceae | *Artemisia* | *Artemisia dubia* |
| Asteraceae | *Artemisia* | *Artemisia eriopoda* |
| Asteraceae | *Artemisia* | *Artemisia japonica* |
| Asteraceae | *Artemisia* | *Artemisia lavandulifolia* |
| Asteraceae | *Artemisia* | *Artemisia scoparia* |
| Asteraceae | *Artemisia* | *Artemisia stechmanniana* |
| Asteraceae | *Artemisia* | *Artemisia vestita* |
| Asteraceae | *Aster* | *Aster ageratoides* |
| Asteraceae | *Aster* | *Aster hispidus* |
| Asteraceae | *Aster* | *Aster indicus* |
| Asteraceae | *Aster* | *Aster lautureanus* |
| Asteraceae | *Aster* | *Aster pekinensis* |
| Asteraceae | *Bidens* | *Bidens bipinnata* |
| Asteraceae | *Bidens* | *Bidens biternata* |
| Asteraceae | *Bidens* | *Bidens parviflora* |
| Asteraceae | *Bidens* | *Bidens pilosa* |
| Asteraceae | *Carduus* | *Carduus crispus* |
| Asteraceae | *Carpesium* | *Carpesium cernuum* |
| Asteraceae | *Chrysanthemum* | *Chrysanthemum chanetii* |
| Asteraceae | *Chrysanthemum* | *Chrysanthemum indicum* |
| Asteraceae | *Chrysanthemum* | *Chrysanthemum lavandulifolium* |
| Asteraceae | *Cichorium* | *Cichorium intybus* |
| Asteraceae | *Cirsium* | *Cirsium arvense* var. *integrifolium* |
| Asteraceae | *Cirsium* | *Cirsium arvense* var. *setosum* |
| Asteraceae | *Cirsium* | *Cirsium chinense* |
| Asteraceae | *Cirsium* | *Cirsium japonicum* |
| Asteraceae | *Cirsium* | *Cirsium maackii* |
| Asteraceae | *Coreopsis* | *Coreopsis basalis* |
| Asteraceae | *Cosmos* | *Cosmos bipinnatus* |
| Asteraceae | *Crepidiastrum* | *Crepidiastrum sonchifolium* |
| Asteraceae | *Dimorphotheca* | *Dimorphotheca ecklonis* |
| Asteraceae | *Echinops* | *Echinops davuricus* |
| Asteraceae | *Echinops* | *Echinops grijsii* |
| Asteraceae | *Eclipta* | *Eclipta prostrata* |
| Asteraceae | *Erigeron* | *Erigeron annuus* |
| Asteraceae | *Erigeron* | *Erigeron bonariensis* |
| Asteraceae | *Erigeron* | *Erigeron canadensis* |
| Asteraceae | *Eupatorium* | *Eupatorium lindleyanum* |
| Asteraceae | *Gaillardia* | *Gaillardia pulchella* |
| Asteraceae | *Helianthus* | *Helianthus tuberosus* |
| Asteraceae | *Hemisteptia* | *Hemisteptia lyrata* |
| Asteraceae | *Inula* | *Inula japonica* |
| Asteraceae | *Inula* | *Inula linariifolia* |
| Asteraceae | *Ixeris* | *Ixeris chinensis* |
| Asteraceae | *Lactuca* | *Lactuca indica* |
| Asteraceae | *Lactuca* | *Lactuca serriola* |
| Asteraceae | *Leibnitzia* | *Leibnitzia anandria* |
| Asteraceae | *Picris* | *Picris hieracioides* |
| Asteraceae | *Saussurea* | *Saussurea japonica* |
| Asteraceae | *Saussurea* | *Saussurea pectinata* |
| Asteraceae | *Scorzonera* | *Scorzonera albicaulis* |
| Asteraceae | *Scorzonera* | *Scorzonera sinensis* |
| Asteraceae | *Solidago* | *Solidago canadensis* |
| Asteraceae | *Sonchus* | *Sonchus asper* |
| Asteraceae | *Sonchus* | *Sonchus brachyotus* |
| Asteraceae | *Sonchus* | *Sonchus oleraceus* |
| Asteraceae | *Sonchus* | *Sonchus wightianus* |
| Asteraceae | *Symphyotrichum* | *Symphyotrichum subulatum* |
| Asteraceae | *Takhtajaniantha* | *Takhtajaniantha austriaca* |
| Asteraceae | *Taraxacum* | *Taraxacum mongolicum* |
| Asteraceae | *Tragopogon* | *Tragopogon pratensis* |
| Asteraceae | *Tripolium* | *Tripolium pannonicum* |
| Asteraceae | *Xanthium* | *Xanthium occidentale* |
| Asteraceae | *Xanthium* | *Xanthium strumarium* |
| Asteraceae | *Xanthium* | *Xanthium strumarium* subsp. *italicum* |
| Basellaceae | *Basella* | *Basella alba* |
| Berberidaceae | *Berberis* | *Berberis thunbergii* cv. *Atropurpurea* |
| Berberidaceae | *Nandina* | *Nandina domestica* |
| Bignoniaceae | *Campsis* | *Campsis grandiflora* |
| Boraginaceae | *Bothriospermum* | *Bothriospermum kusnetzowii* |
| Boraginaceae | *Bothriospermum* | *Bothriospermum secundum* |
| Boraginaceae | *Lappula* | *Lappula myosotis* |
| Boraginaceae | *Lithospermum* | *Lithospermum arvense* |
| Boraginaceae | *Thyrocarpus* | *Thyrocarpus glochidiatus* |
| Boraginaceae | *Tournefortia* | *Tournefortia sibirica* |
| Boraginaceae | *Trigonotis* | *Trigonotis peduncularis* |
| Brassicaceae | *Capsella* | *Capsella bursa-pastoris* |
| Brassicaceae | *Cardamine* | *Cardamine hirsuta* |
| Brassicaceae | *Cardamine* | *Cardamine occulta* |
| Brassicaceae | *Descurainia* | *Descurainia sophia* |
| Brassicaceae | *Lepidium* | *Lepidium virginicum* |
| Brassicaceae | *Orychophragmus* | *Orychophragmus violaceus* |
| Brassicaceae | *Rorippa* | *Rorippa palustris* |
| Brassicaceae | *Thlaspi* | *Thlaspi arvense* |
| Buxaceae | *Buxus* | *Buxus sinica* var. *parvifolia* |
| Campanulaceae | *Adenophora* | *Adenophora polyantha* |
| Campanulaceae | *Adenophora* | *Adenophora trachelioides* |
| Cannabaceae | *Cannabis* | *Cannabis sativa* |
| Cannabaceae | *Celtis* | *Celtis bungeana* |
| Cannabaceae | *Humulus* | *Humulus scandens* |
| Caprifoliaceae | *Lonicera* | *Lonicera japonica* |
| Caprifoliaceae | *Weigela* | *Weigela florida* |
| Caryophyllaceae | *Dianthus* | *Dianthus chinensis* |
| Caryophyllaceae | *Gypsophila* | *Gypsophila oldhamiana* |
| Caryophyllaceae | *Sagina* | *Sagina japonica* |
| Caryophyllaceae | *Stellaria* | *Stellaria aquatica* |
| Caryophyllaceae | *Stellaria* | *Stellaria dichotoma* |
| Caryophyllaceae | *Stellaria* | *Stellaria media* |
| Caryophyllaceae | *Stellaria* | *Stellaria pallida* |
| Celastraceae | *Celastrus* | *Celastrus orbiculatus* |
| Celastraceae | *Euonymus* | *Euonymus alatus* |
| Celastraceae | *Euonymus* | *Euonymus fortunei* |
| Celastraceae | *Euonymus* | *Euonymus japonicus* |
| Celastraceae | *Euonymus* | *Euonymus maackii* |
| Commelinaceae | *Commelina* | *Commelina benghalensis* |
| Commelinaceae | *Commelina* | *Commelina communis* |
| Convolvulaceae | *Calystegia* | *Calystegia hederacea* |
| Convolvulaceae | *Calystegia* | *Calystegia pellita* |
| Convolvulaceae | *Calystegia* | *Calystegia sepium* |
| Convolvulaceae | *Calystegia* | *Calystegia soldanella* |
| Convolvulaceae | *Convolvulus* | *Convolvulus ammannii* |
| Convolvulaceae | *Convolvulus* | *Convolvulus arvensis* |
| Convolvulaceae | *Ipomoea* | *Ipomoea nil* |
| Convolvulaceae | *Ipomoea* | *Ipomoea purpurea* |
| Convolvulaceae | *Merremia* | *Merremia sibirica* |
| Crassulaceae | *Hylotelephium* | *Hylotelephium malacophyllum* |
| Crassulaceae | *Hylotelephium* | *Hylotelephium spectabile* |
| Crassulaceae | *Orostachys* | *Orostachys fimbriata* |
| Crassulaceae | *Orostachys* | *Orostachys japonica* |
| Crassulaceae | *Phedimus* | *Phedimus aizoon* |
| Crassulaceae | *Phedimus* | *Phedimus kamtschaticus* |
| Cucurbitaceae | *Trichosanthes* | *Trichosanthes kirilowii* |
| Cupressaceae | *Juniperus* | *Juniperus chinensis* |
| Cupressaceae | *Juniperus* | *Juniperus chinensis* cv. *Kaizuca* |
| Cupressaceae | *Metasequoia* | *Metasequoia glyptostroboides* |
| Cupressaceae | *Platycladus* | *Platycladus orientalis* |
| Cyperaceae | *Carex* | *Carex breviculmis* |
| Cyperaceae | *Carex* | *Carex duriuscula* subsp. *rigescens* |
| Cyperaceae | *Carex* | *Carex heterostachya* |
| Cyperaceae | *Carex* | *Carex lanceolata* |
| Cyperaceae | *Cyperus* | *Cyperus glomeratus* |
| Cyperaceae | *Cyperus* | *Cyperus microiria* |
| Dioscoreaceae | *Dioscorea* | *Dioscorea nipponica* |
| Dioscoreaceae | *Dioscorea* | *Dioscorea polystachya* |
| Dryopteridaceae | *Cyrtomium* | *Cyrtomium falcatum* |
| Ebenaceae | *Diospyros* | *Diospyros kaki* |
| Ebenaceae | *Diospyros* | *Diospyros lotus* |
| Elaeagnaceae | *Elaeagnus* | *Elaeagnus umbellata* |
| Ephedraceae | *Ephedra* | *Ephedra sinica* |
| Equisetaceae | *Equisetum* | *Equisetum ramosissimum* |
| Ericaceae | *Rhododendron* | *Rhododendron micranthum* |
| Euphorbiaceae | *Acalypha* | *Acalypha australis* |
| Euphorbiaceae | *Euphorbia* | *Euphorbia esula* |
| Euphorbiaceae | *Euphorbia* | *Euphorbia fischeriana* |
| Euphorbiaceae | *Euphorbia* | *Euphorbia helioscopia* |
| Euphorbiaceae | *Euphorbia* | *Euphorbia humifusa* |
| Euphorbiaceae | *Euphorbia* | *Euphorbia maculata* |
| Euphorbiaceae | *Euphorbia* | *Euphorbia pekinensis* |
| Euphorbiaceae | *Euphorbia* | *Euphorbia prostrata* |
| Euphorbiaceae | *Speranskia* | *Speranskia tuberculata* |
| Fabaceae | *Albizia* | *Albizia julibrissin* |
| Fabaceae | *Albizia* | *Albizia kalkora* |
| Fabaceae | *Amorpha* | *Amorpha fruticosa* |
| Fabaceae | *Amphicarpaea* | *Amphicarpaea edgeworthii* |
| Fabaceae | *Astragalus* | *Astragalus dahuricus* |
| Fabaceae | *Astragalus* | *Astragalus scaberrimus* |
| Fabaceae | *Caragana* | *Caragana leveillei* |
| Fabaceae | *Caragana* | *Caragana rosea* |
| Fabaceae | *Caragana* | *Caragana sinica* |
| Fabaceae | *Cercis* | *Cercis chinensis* |
| Fabaceae | *Chamaecrista* | *Chamaecrista nomame* |
| Fabaceae | *Gleditsia* | *Gleditsia japonica* |
| Fabaceae | *Gleditsia* | *Gleditsia sinensis* |
| Fabaceae | *Gueldenstaedtia* | *Gueldenstaedtia verna* |
| Fabaceae | *Indigofera* | *Indigofera bungeana* |
| Fabaceae | *Indigofera* | *Indigofera kirilowii* |
| Fabaceae | *Kummerowia* | *Kummerowia striata* |
| Fabaceae | *Lespedeza* | *Lespedeza bicolor* |
| Fabaceae | *Lespedeza* | *Lespedeza caraganae* |
| Fabaceae | *Lespedeza* | *Lespedeza cuneata* |
| Fabaceae | *Lespedeza* | *Lespedeza davurica* |
| Fabaceae | *Lespedeza* | *Lespedeza floribunda* |
| Fabaceae | *Lespedeza* | *Lespedeza juncea* |
| Fabaceae | *Lespedeza* | *Lespedeza potaninii* |
| Fabaceae | *Lespedeza* | *Lespedeza tomentosa* |
| Fabaceae | *Medicago* | *Medicago lupulina* |
| Fabaceae | *Medicago* | *Medicago sativa* |
| Fabaceae | *Melilotus* | *Melilotus albus* |
| Fabaceae | *Melilotus* | *Melilotus officinalis* |
| Fabaceae | *Pueraria* | *Pueraria montana* var. *lobata* |
| Fabaceae | *Robinia* | *Robinia pseudoacacia* |
| Fabaceae | *Sophora* | *Sophora flavescens* |
| Fabaceae | *Styphnolobium* | *Styphnolobium japonicum* |
| Fabaceae | *Trifolium* | *Trifolium repens* |
| Fabaceae | *Vicia* | *Vicia amoena* |
| Fabaceae | *Vicia* | *Vicia bungei* |
| Fabaceae | *Vicia* | *Vicia cracca* |
| Fabaceae | *Vigna* | *Vigna minima* |
| Fabaceae | *Vigna* | *Vigna radiata* var. *sublobata* |
| Fabaceae | *Wisteria* | *Wisteria sinensis* |
| Fagaceae | *Quercus* | *Quercus acutissima* |
| Fagaceae | *Quercus* | *Quercus dentata* |
| Fagaceae | *Quercus* | *Quercus mongolica* |
| Fagaceae | *Quercus* | *Quercus variabilis* |
| Geraniaceae | *Erodium* | *Erodium stephanianum* |
| Geraniaceae | *Geranium* | *Geranium sibiricum* |
| Ginkgoaceae | *Ginkgo* | *Ginkgo biloba* |
| Hydrangeaceae | *Deutzia* | *Deutzia grandiflora* |
| Iridaceae | *Belamcanda* | *Belamcanda chinensis* |
| Iridaceae | *Iris* | *Iris dichotoma* |
| Iridaceae | *Iris* | *Iris lactea* |
| Iridaceae | *Iris* | *Iris tectorum* |
| Juglandaceae | *Juglans* | *Juglans regia* |
| Juncaceae | *Juncus* | *Juncus effusus* |
| Lamiaceae | *Ajuga* | *Ajuga linearifolia* |
| Lamiaceae | *Ajuga* | *Ajuga multiflora* |
| Lamiaceae | *Clerodendrum* | *Clerodendrum trichotomum* |
| Lamiaceae | *Lagopsis* | *Lagopsis supina* |
| Lamiaceae | *Leonurus* | *Leonurus japonicus* |
| Lamiaceae | *Mentha* | *Mentha canadensis* |
| Lamiaceae | *Perilla* | *Perilla frutescens* |
| Lamiaceae | *Salvia* | *Salvia nemorosa* |
| Lamiaceae | *Salvia* | *Salvia plebeia* |
| Lamiaceae | *Scutellaria* | *Scutellaria baicalensis* |
| Lamiaceae | *Teucrium* | *Teucrium ussuriense* |
| Lamiaceae | *Teucrium* | *Teucrium viscidum* |
| Lamiaceae | *Thymus* | *Thymus mongolicus* |
| Lamiaceae | *Vitex* | *Vitex negundo* |
| Lamiaceae | *Vitex* | *Vitex negundo* var. *heterophylla* |
| Liliaceae | *Lilium* | *Lilium lancifolium* |
| Liliaceae | *Lilium* | *Lilium pumilum* |
| Lythraceae | *Lagerstroemia* | *Lagerstroemia indica* |
| Lythraceae | *Punica* | *Punica granatum* |
| Magnoliaceae | *Magnolia* | *Magnolia grandiflora* |
| Malvaceae | *Abutilon* | *Abutilon theophrasti* |
| Malvaceae | *Firmiana* | *Firmiana simplex* |
| Malvaceae | *Grewia* | *Grewia biloba* |
| Malvaceae | *Hibiscus* | *Hibiscus syriacus* |
| Malvaceae | *Hibiscus* | *Hibiscus trionum* |
| Malvaceae | *Malva* | *Malva pusilla* |
| Malvaceae | *Malva* | *Malva verticillata* var. *crispa* |
| Mazaceae | *Mazus* | *Mazus pumilus* |
| Meliaceae | *Melia* | *Melia azedarach* |
| Meliaceae | *Toona* | *Toona sinensis* |
| Menispermaceae | *Cocculus* | *Cocculus orbiculatus* |
| Moraceae | *Broussonetia* | *Broussonetia papyrifera* |
| Moraceae | *Ficus* | *Ficus carica* |
| Moraceae | *Maclura* | *Maclura tricuspidata* |
| Moraceae | *Morus* | *Morus alba* |
| Moraceae | *Morus* | *Morus mongolica* |
| Oleaceae | *Fontanesia* | *Fontanesia philliraeoides* var. *fortunei* |
| Oleaceae | *Forsythia* | *Forsythia suspensa* |
| Oleaceae | *Forsythia* | *Forsythia viridissima* |
| Oleaceae | *Fraxinus* | *Fraxinus chinensis* |
| Oleaceae | *Fraxinus* | *Fraxinus pennsylvanica* |
| Oleaceae | *Fraxinus* | *Fraxinus xanthoxyloides* |
| Oleaceae | *Jasminum* | *Jasminum nudiflorum* |
| Oleaceae | *Ligustrum* | *Ligustrum lucidum* |
| Oleaceae | *Ligustrum* | *Ligustrum quihoui* |
| Oleaceae | *Syringa* | *Syringa pubescens* subsp. *microphylla* |
| Oleaceae | *Syringa* | *Syringa vulgaris* |
| Onagraceae | *Gaura* | *Gaura parviflora* |
| Onagraceae | *Oenothera* | *Oenothera biennis* |
| Orobanchaceae | *Rehmannia* | *Rehmannia glutinosa* |
| Orobanchaceae | *Siphonostegia* | *Siphonostegia chinensis* |
| Oxalidaceae | *Oxalis* | *Oxalis corniculata* |
| Papaveraceae | *Corydalis* | *Corydalis bungeana* |
| Papaveraceae | *Corydalis* | *Corydalis caudata* |
| Paulowniaceae | *Paulownia* | *Paulownia catalpifolia* |
| Paulowniaceae | *Paulownia* | *Paulownia tomentosa* |
| Phrymaceae | *Phryma* | *Phryma leptostachya* subsp. *asiatica* |
| Phyllanthaceae | *Flueggea* | *Flueggea suffruticosa* |
| Phytolaccaceae | *Phytolacca* | *Phytolacca americana* |
| Pinaceae | *Cedrus* | *Cedrus deodara* |
| Pinaceae | *Picea* | *Picea wilsonii* |
| Pinaceae | *Pinus* | *Pinus densiflora* |
| Pinaceae | *Pinus* | *Pinus thunbergii* |
| Plantaginaceae | *Plantago* | *Plantago asiatica* |
| Plantaginaceae | *Plantago* | *Plantago depressa* |
| Plantaginaceae | *Plantago* | *Plantago lanceolata* |
| Plantaginaceae | *Plantago* | *Plantago major* |
| Plantaginaceae | *Pseudolysimachion* | *Pseudolysimachion linariifolium* |
| Plantaginaceae | *Veronica* | *Veronica persica* |
| Plantaginaceae | *Veronica* | *Veronica polita* |
| Platanaceae | *Platanus* | *Platanus acerifolia* |
| Platanaceae | *Platanus* | *Platanus occidentalis* |
| Plumbaginaceae | *Limonium* | *Limonium bicolor* |
| Plumbaginaceae | *Limonium* | *Limonium franchetii* |
| Poaceae | *Achnatherum* | *Achnatherum pekinense* |
| Poaceae | *Arthraxon* | *Arthraxon hispidus* |
| Poaceae | *Arthraxon* | *Arthraxon prionodes* |
| Poaceae | *Arundinella* | *Arundinella hirta* |
| Poaceae | *Arundo* | *Arundo donax* |
| Poaceae | *Bothriochloa* | *Bothriochloa ischaemum* |
| Poaceae | *Bromus* | *Bromus japonicus* |
| Poaceae | *Calamagrostis* | *Calamagrostis epigeios* |
| Poaceae | *Calamagrostis* | *Calamagrostis pseudophragmites* |
| Poaceae | *Capillipedium* | *Capillipedium parviflorum* |
| Poaceae | *Chloris* | *Chloris virgata* |
| Poaceae | *Cleistogenes* | *Cleistogenes hackelii* |
| Poaceae | *Cleistogenes* | *Cleistogenes polyphylla* |
| Poaceae | *Cymbopogon* | *Cymbopogon goeringii* |
| Poaceae | *Cynodon* | *Cynodon dactylon* |
| Poaceae | *Deyeuxia* | *Deyeuxia pyramidalis* |
| Poaceae | *Digitaria* | *Digitaria ciliaris* var. *chrysoblephara* |
| Poaceae | *Digitaria* | *Digitaria sanguinalis* |
| Poaceae | *Echinochloa* | *Echinochloa colona* |
| Poaceae | *Eleusine* | *Eleusine indica* |
| Poaceae | *Elymus* | *Elymus ciliaris* |
| Poaceae | *Elymus* | *Elymus dahuricus* |
| Poaceae | *Elymus* | *Elymus kamoji* |
| Poaceae | *Eragrostis* | *Eragrostis cilianensis* |
| Poaceae | *Eragrostis* | *Eragrostis minor* |
| Poaceae | *Eragrostis* | *Eragrostis pilosa* |
| Poaceae | *Hemarthria* | *Hemarthria altissima* |
| Poaceae | *Hordeum* | *Hordeum jubatum* |
| Poaceae | *Imperata* | *Imperata cylindrica* |
| Poaceae | *Koeleria* | *Koeleria macrantha* |
| Poaceae | *Leymus* | *Leymus chinensis* |
| Poaceae | *Lolium* | *Lolium perenne* |
| Poaceae | *Lolium* | *Lolium persicum* |
| Poaceae | *Melica* | *Melica scabrosa* |
| Poaceae | *Miscanthus* | *Miscanthus sacchariflorus* |
| Poaceae | *Miscanthus* | *Miscanthus sinensis* |
| Poaceae | *Muhlenbergia* | *Muhlenbergia capillaris* |
| Poaceae | *Muhlenbergia* | *Muhlenbergia huegelii* |
| Poaceae | *Oplismenus* | *Oplismenus undulatifolius* |
| Poaceae | *Panicum* | *Panicum bisulcatum* |
| Poaceae | *Panicum* | *Panicum elegantissimum* |
| Poaceae | *Panicum* | *Panicum virgatum* |
| Poaceae | *Pennisetum* | *Pennisetum alopecuroides* |
| Poaceae | *Phragmites* | *Phragmites australis* |
| Poaceae | *Phyllostachys* | *Phyllostachys propinqua* |
| Poaceae | *Poa* | *Poa pratensis* |
| Poaceae | *Setaria* | *Setaria faberi* |
| Poaceae | *Setaria* | *Setaria pumila* |
| Poaceae | *Setaria* | *Setaria viridis* |
| Poaceae | *Spodiopogon* | *Spodiopogon sibiricus* |
| Poaceae | *Themeda* | *Themeda triandra* |
| Poaceae | *Zoysia* | *Zoysia japonica* |
| Poaceae | *Zoysia* | *Zoysia pacifica* |
| Polygalaceae | *Polygala* | *Polygala tenuifolia* |
| Polygonaceae | *Fallopia* | *Fallopia dentatoalata* |
| Polygonaceae | *Koenigia* | *Koenigia divaricata* |
| Polygonaceae | *Persicaria* | *Persicaria bungeana* |
| Polygonaceae | *Pleuropterus* | *Pleuropterus multiflorus* |
| Polygonaceae | *Polygonum* | *Polygonum aviculare* |
| Polygonaceae | *Polygonum* | *Polygonum plebeium* |
| Polygonaceae | *Reynoutria* | *Reynoutria japonica* |
| Polygonaceae | *Rumex* | *Rumex crispus* |
| Polygonaceae | *Rumex* | *Rumex dentatus* |
| Polygonaceae | *Rumex* | *Rumex japonicus* |
| Polygonaceae | *Rumex* | *Rumex patientia* |
| Portulacaceae | *Portulaca* | *Portulaca oleracea* |
| Primulaceae | *Lysimachia* | *Lysimachia barystachys* |
| Primulaceae | *Lysimachia* | *Lysimachia clethroides* |
| Primulaceae | *Lysimachia* | *Lysimachia pentapetala* |
| Ranunculaceae | *Clematis* | *Clematis heracleifolia* |
| Ranunculaceae | *Clematis* | *Clematis hexapetala* |
| Ranunculaceae | *Pulsatilla* | *Pulsatilla chinensis* |
| Ranunculaceae | *Thalictrum* | *Thalictrum minus* var. *hypoleucum* |
| Rhamnaceae | *Frangula* | *Frangula crenata* |
| Rhamnaceae | *Rhamnella* | *Rhamnella franguloides* |
| Rhamnaceae | *Rhamnus* | *Rhamnus globosa* |
| Rhamnaceae | *Rhamnus* | *Rhamnus parvifolia* |
| Rhamnaceae | *Ziziphus* | *Ziziphus jujuba* var. *spinosa* |
| Rosaceae | *Agrimonia* | *Agrimonia pilosa* |
| Rosaceae | *Cotoneaster* | *Cotoneaster multiflorus* |
| Rosaceae | *Crataegus* | *Crataegus pinnatifida* |
| Rosaceae | *Fragaria* | *Fragaria × ananassa* |
| Rosaceae | *Kerria* | *Kerria japonica* |
| Rosaceae | *Malus* | *Malus × micromalus* |
| Rosaceae | *Malus* | *Malus pumila* |
| Rosaceae | *Photinia* | *Photinia × fraseri* |
| Rosaceae | *Potentilla* | *Potentilla chinensis* |
| Rosaceae | *Potentilla* | *Potentilla supina* |
| Rosaceae | *Potentilla* | *Potentilla tanacetifolia* |
| Rosaceae | *Prunus* | *Prunus cerasifera* cv. *Atropurpurea* |
| Rosaceae | *Prunus* | *Prunus davidiana* |
| Rosaceae | *Prunus* | *Prunus humilis* |
| Rosaceae | *Prunus* | *Prunus serrulata* var. *lannesiana* |
| Rosaceae | *Prunus* | *Prunus sibirica* |
| Rosaceae | *Prunus* | *Prunus tomentosa* |
| Rosaceae | *Prunus* | *Prunus triloba* |
| Rosaceae | *Pyracantha* | *Pyracantha fortuneana* |
| Rosaceae | *Pyrus* | *Pyrus calleryana* |
| Rosaceae | *Rosa* | *Rosa chinensis* |
| Rosaceae | *Rosa* | *Rosa multiflora* |
| Rosaceae | *Rosa* | *Rosa rugosa* |
| Rosaceae | *Rubus* | *Rubus parvifolius* |
| Rosaceae | *Sanguisorba* | *Sanguisorba officinalis* |
| Rosaceae | *Spiraea* | *Spiraea trilobata* |
| Rubiaceae | *Galium* | *Galium spurium* |
| Rubiaceae | *Galium* | *Galium verum* |
| Rubiaceae | *Rubia* | *Rubia cordifolia* |
| Rubiaceae | *Rubia* | *Rubia sylvatica* |
| Rubiaceae | *Rubia* | *Rubia truppeliana* |
| Rutaceae | *Tetradium* | *Tetradium daniellii* |
| Rutaceae | *Zanthoxylum* | *Zanthoxylum bungeanum* |
| Rutaceae | *Zanthoxylum* | *Zanthoxylum schinifolium* |
| Rutaceae | *Zanthoxylum* | *Zanthoxylum simulans* |
| Salicaceae | *Populus* | *Populus × beijingensis* |
| Salicaceae | *Populus* | *Populus alba* |
| Salicaceae | *Populus* | *Populus tomentosa* |
| Salicaceae | *Salix* | *Salix matsudana* |
| Sapindaceae | *Acer* | *Acer negundo* |
| Sapindaceae | *Acer* | *Acer saccharinum* |
| Sapindaceae | *Acer* | *Acer truncatum* |
| Sapindaceae | *Koelreuteria* | *Koelreuteria bipinnata* |
| Sapindaceae | *Koelreuteria* | *Koelreuteria paniculata* |
| Sapindaceae | *Xanthoceras* | *Xanthoceras sorbifolium* |
| Simaroubaceae | *Ailanthus* | *Ailanthus altissima* |
| Simaroubaceae | *Picrasma* | *Picrasma quassioides* |
| Smilacaceae | *Smilax* | *Smilax riparia* |
| Smilacaceae | *Smilax* | *Smilax sieboldii* |
| Solanaceae | *Datura* | *Datura innoxia* |
| Solanaceae | *Datura* | *Datura stramonium* |
| Solanaceae | *Lycium* | *Lycium chinense* |
| Solanaceae | *Solanum* | *Solanum lyratum* |
| Solanaceae | *Solanum* | *Solanum nigrum* |
| Tamaricaceae | *Tamarix* | *Tamarix chinensis* |
| Ulmaceae | *Hemiptelea* | *Hemiptelea davidii* |
| Ulmaceae | *Ulmus* | *Ulmus davidiana* var. *japonica* |
| Ulmaceae | *Ulmus* | *Ulmus macrocarpa* |
| Ulmaceae | *Ulmus* | *Ulmus parvifolia* |
| Ulmaceae | *Ulmus* | *Ulmus pumila* |
| Viburnaceae | *Sambucus* | *Sambucus williamsii* |
| Violaceae | *Viola* | *Viola phalacrocarpa* |
| Violaceae | *Viola* | *Viola prionantha* |
| Vitaceae | *Ampelopsis* | *Ampelopsis humulifolia* |
| Vitaceae | *Ampelopsis* | *Ampelopsis japonica* |
| Vitaceae | *Causonis* | *Causonis japonica* |
| Vitaceae | *Parthenocissus* | *Parthenocissus quinquefolia* |
| Vitaceae | *Parthenocissus* | *Parthenocissus tricuspidata* |
| Vitaceae | *Vitis* | *Vitis amurensis* |
| Zygophyllaceae | *Tribulus* | *Tribulus terrestris* |

**Table S3.** Richness of all plants and different life forms on each island.

| **Island name** | **All plants** | **Tree** | **Shrub** | **Herb** | **Invasive plants** |
| --- | --- | --- | --- | --- | --- |
| Beihuangcheng Island | 164 | 14 | 29 | 121 | 13 |
| Nanhuangcheng Island | 158 | 15 | 37 | 106 | 18 |
| Daqin Island | 201 | 20 | 35 | 146 | 14 |
| Xiaoqin Island | 88 | 10 | 18 | 60 | 4 |
| Daheishan Island | 223 | 24 | 29 | 170 | 21 |
| Xiaoheishan Island | 84 | 16 | 14 | 54 | 4 |
| Beichangshan Island | 204 | 26 | 31 | 147 | 23 |
| Nanchangshan Island | 210 | 33 | 39 | 138 | 25 |
| Tuoji Island | 226 | 28 | 49 | 149 | 17 |
| Miaodao Island | 84 | 14 | 13 | 57 | 10 |

**Table S4.** Diversity index of all plants and different life forms on each island.

|  | Shannon-Wiener diversity index | Pielou evenness index | Simpson diversity index |
| --- | --- | --- | --- |
| **All plants** |  |  |  |
| Beihuangcheng Island | 1.64 | 0.41 | 0.57 |
| Beichangshan Island | 1.44 | 0.36 | 0.50 |
| Daheishan Island | 3.13 | 0.73 | 0.92 |
| Daqin Island | 1.72 | 0.42 | 0.63 |
| Miaodao Island | 1.91 | 0.53 | 0.65 |
| Nanhuangcheng Island | 1.92 | 0.47 | 0.65 |
| Nanchangshan Island | 1.40 | 0.35 | 0.49 |
| Tuoji Island | 2.53 | 0.59 | 0.85 |
| Xiaoheishan Island | 2.18 | 0.58 | 0.80 |
| Xiaoqin Island | 1.90 | 0.51 | 0.71 |
| **Tree** |  |  |  |
| Beihuangcheng Island | 0.39 | 0.24 | 0.16 |
| Beichangshan Island | 1.16 | 0.53 | 0.57 |
| Daheishan Island | 1.78 | 0.72 | 0.79 |
| Daqin Island | 0.64 | 0.33 | 0.30 |
| Miaodao Island | 1.23 | 0.63 | 0.64 |
| Nanhuangcheng Island | 1.28 | 0.66 | 0.68 |
| Nanchangshan Island | 1.18 | 0.51 | 0.62 |
| Tuoji Island | 1.04 | 0.50 | 0.58 |
| Xiaoheishan Island | 1.11 | 0.62 | 0.59 |
| Xiaoqin Island | 0.25 | 0.16 | 0.09 |
| **Shrub** |  |  |  |
| Beihuangcheng Island | 1.91 | 0.64 | 0.77 |
| Beichangshan Island | 1.60 | 0.56 | 0.65 |
| Daheishan Island | 2.33 | 0.76 | 0.87 |
| Daqin Island | 1.98 | 0.69 | 0.80 |
| Miaodao Island | 2.19 | 0.81 | 0.86 |
| Nanhuangcheng Island | 2.16 | 0.82 | 0.82 |
| Nanchangshan Island | 2.23 | 0.77 | 0.86 |
| Tuoji Island | 2.41 | 0.73 | 0.85 |
| Xiaoheishan Island | 2.07 | 0.83 | 0.84 |
| Xiaoqin Island | 2.27 | ] | 0.87 |
| **Herb** |  |  |  |
| Beihuangcheng Island | 1.32 | 0.37 | 0.50 |
| Beichangshan Island | 1.12 | 0.31 | 0.41 |
| Daheishan Island | 2.69 | 0.69 | 0.88 |
| Daqin Island | 1.47 | 0.40 | 0.58 |
| Miaodao Island | 1.37 | 0.45 | 0.52 |
| Nanhuangcheng Island | 1.60 | 0.43 | 0.58 |
| Nanchangshan Island | 1.12 | 0.31 | 0.42 |
| Tuoji Island | 2.27 | 0.59 | 0.83 |
| Xiaoheishan Island | 1.87 | 0.55 | 0.76 |
| Xiaoqin Island | 1.47 | 0.47 | 0.64 |

**Table S5.** Jaccard index (J) between each pair of two islands for all plants, tree, shrub, herb species. J, J1, J2, J3 and J4 are Jaccard indices for all plants, tree, shrub, herb, invasive plants, respectively.

| Island | Species | Beihuangcheng Island | Nanhuangcheng Island | Daqin Island | Xiaoqin Island | Daheishan Island | Xiaoheishan Island | Beichangshan Island | Nanchangshan Island | Tuoji Island | Miaodao Island |
| --- | --- | --- | --- | --- | --- | --- | --- | --- | --- | --- | --- |
| Beihuangcheng Island | J | - | 0.543 | 0.534 | 0.660 | 0.613 | 0.715 | 0.671 | 0.697 | 0.566 | 0.741 |
|  | J1 | - | 0.619 | 0.640 | 0.500 | 0.643 | 0.571 | 0.824 | 0.795 | 0.600 | 0.600 |
|  | J2 | - | 0.625 | 0.609 | 0.618 | 0.739 | 0.735 | 0.723 | 0.764 | 0.607 | 0.765 |
|  | J3 | - | 0.507 | 0.500 | 0.688 | 0.580 | 0.732 | 0.633 | 0.658 | 0.548 | 0.755 |
|  | J4 | - | 0.591 | 0.412 | 0.867 | 0.522 | 0.786 | 0.615 | 0.690 | 0.571 | 0.722 |
| Nanhuangcheng Island | J | 0.543 | - | 0.575 | 0.633 | 0.677 | 0.720 | 0.649 | 0.718 | 0.629 | 0.746 |
|  | J1 | 0.619 | - | 0.542 | 0.333 | 0.655 | 0.652 | 0.719 | 0.829 | 0.735 | 0.550 |
|  | J2 | 0.625 | - | 0.588 | 0.721 | 0.653 | 0.725 | 0.612 | 0.643 | 0.491 | 0.718 |
|  | J3 | 0.507 | - | 0.576 | 0.639 | 0.686 | 0.730 | 0.647 | 0.716 | 0.651 | 0.784 |
|  | J4 | 0.591 | - | 0.545 | 0.900 | 0.700 | 0.900 | 0.633 | 0.735 | 0.600 | 0.727 |
| Daqin Island | J | 0.534 | 0.575 | - | 0.686 | 0.650 | 0.766 | 0.672 | 0.712 | 0.586 | 0.782 |
|  | J1 | 0.640 | 0.542 | - | 0.571 | 0.667 | 0.615 | 0.757 | 0.767 | 0.667 | 0.522 |
|  | J2 | 0.609 | 0.588 | - | 0.707 | 0.638 | 0.775 | 0.625 | 0.655 | 0.526 | 0.769 |
|  | J3 | 0.500 | 0.576 | - | 0.696 | 0.650 | 0.788 | 0.668 | 0.715 | 0.589 | 0.820 |
|  | J4 | 0.412 | 0.900 | - | 0.800 | 0.542 | 0.800 | 0.577 | 0.655 | 0.591 | 0.737 |
| Xiaoqin Island | J | 0.660 | 0.633 | 0.686 | - | 0.736 | 0.735 | 0.730 | 0.763 | 0.713 | 0.697 |
|  | J1 | 0.500 | 0.333 | 0.571 | - | 0.692 | 0.700 | 0.759 | 0.838 | 0.733 | 0.500 |
|  | J2 | 0.618 | 0.721 | 0.707 | - | 0.657 | 0.769 | 0.676 | 0.705 | 0.759 | 0.708 |
|  | J3 | 0.688 | 0.639 | 0.696 | - | 0.757 | 0.733 | 0.738 | 0.763 | 0.694 | 0.728 |
|  | J4 | 0.867 | 0.900 | 0.800 | - | 0.810 | 0.857 | 0.875 | 0.840 | 0.895 | 0.600 |
| Daheishan Island | J | 0.613 | 0.677 | 0.650 | 0.736 | - | 0.762 | 0.605 | 0.625 | 0.610 | 0.767 |
|  | J1 | 0.643 | 0.655 | 0.667 | 0.692 | - | 0.621 | 0.611 | 0.643 | 0.556 | 0.593 |
|  | J2 | 0.739 | 0.653 | 0.638 | 0.657 | - | 0.771 | 0.636 | 0.717 | 0.655 | 0.727 |
|  | J3 | 0.580 | 0.686 | 0.650 | 0.757 | - | 0.783 | 0.597 | 0.600 | 0.607 | 0.799 |
|  | J4 | 0.522 | 0.700 | 0.542 | 0.810 | - | 0.810 | 0.581 | 0.647 | 0.733 | 0.760 |
| Xiaoheishan Island | J | 0.715 | 0.720 | 0.766 | 0.735 | 0.762 | - | 0.769 | 0.759 | 0.750 | 0.708 |
|  | J1 | 0.571 | 0.652 | 0.615 | 0.700 | 0.621 | - | 0.765 | 0.711 | 0.581 | 0.500 |
|  | J2 | 0.735 | 0.725 | 0.775 | 0.769 | 0.771 | - | 0.714 | 0.767 | 0.765 | 0.650 |
|  | J3 | 0.732 | 0.730 | 0.788 | 0.733 | 0.783 | - | 0.782 | 0.769 | 0.777 | 0.767 |
|  | J4 | 0.786 | 0.900 | 0.800 | 0.857 | 0.810 | - | 0.875 | 0.840 | 0.895 | 0.923 |
| Beichangshan Island | J | 0.671 | 0.649 | 0.672 | 0.730 | 0.605 | 0.769 | - | 0.615 | 0.639 | 0.764 |
|  | J1 | 0.824 | 0.719 | 0.757 | 0.759 | 0.611 | 0.765 | - | 0.689 | 0.650 | 0.710 |
|  | J2 | 0.723 | 0.612 | 0.625 | 0.676 | 0.636 | 0.714 | - | 0.571 | 0.596 | 0.706 |
|  | J3 | 0.633 | 0.647 | 0.668 | 0.738 | 0.597 | 0.782 | - | 0.610 | 0.648 | 0.786 |
|  | J4 | 0.615 | 0.633 | 0.577 | 0.875 | 0.581 | 0.875 | - | 0.629 | 0.667 | 0.778 |
| Nanchangshan Island | J | 0.697 | 0.718 | 0.712 | 0.763 | 0.625 | 0.759 | 0.615 | - | 0.629 | 0.754 |
|  | J1 | 0.795 | 0.829 | 0.767 | 0.838 | 0.643 | 0.711 | 0.689 | - | 0.644 | 0.694 |
|  | J2 | 0.764 | 0.643 | 0.655 | 0.705 | 0.717 | 0.767 | 0.571 | - | 0.625 | 0.732 |
|  | J3 | 0.658 | 0.716 | 0.715 | 0.763 | 0.600 | 0.769 | 0.610 | - | 0.627 | 0.774 |
|  | J4 | 0.690 | 0.735 | 0.655 | 0.840 | 0.647 | 0.840 | 0.629 | - | 0.645 | 0.704 |
| Tuoji Island | J | 0.566 | 0.629 | 0.586 | 0.713 | 0.610 | 0.750 | 0.639 | 0.629 | - | 0.775 |
|  | J1 | 0.600 | 0.735 | 0.667 | 0.733 | 0.556 | 0.581 | 0.650 | 0.644 | - | 0.688 |
|  | J2 | 0.607 | 0.491 | 0.526 | 0.759 | 0.655 | 0.765 | 0.596 | 0.625 | - | 0.760 |
|  | J3 | 0.548 | 0.651 | 0.589 | 0.694 | 0.607 | 0.777 | 0.648 | 0.627 | - | 0.795 |
|  | J4 | 0.571 | 0.600 | 0.591 | 0.895 | 0.733 | 0.895 | 0.667 | 0.645 | - | 0.773 |
| Miaodao Island | J | 0.741 | 0.746 | 0.782 | 0.697 | 0.767 | 0.708 | 0.764 | 0.754 | 0.775 | - |
|  | J1 | 0.600 | 0.550 | 0.522 | 0.500 | 0.593 | 0.500 | 0.710 | 0.694 | 0.688 | - |
|  | J2 | 0.765 | 0.718 | 0.769 | 0.708 | 0.727 | 0.650 | 0.706 | 0.732 | 0.760 | - |
|  | J3 | 0.755 | 0.784 | 0.820 | 0.728 | 0.799 | 0.767 | 0.786 | 0.774 | 0.795 | - |
|  | J4 | 0.722 | 0.727 | 0.737 | 0.600 | 0.760 | 0.923 | 0.778 | 0.704 | 0.773 | - |

**Table S6.** Generalised linear modelling assesses the impact and significance of various environmental factors on plant richness.

|  | **Estimate** | **Std.Error** | **z-value** | **Pr(>\|z\|)** |
| --- | --- | --- | --- | --- |
| **All plants** |  |  |  |  |
| A | 0.409 | 0.057 | 7.127 | <0.001 |
| DM | -0.157 | 0.073 | -2.142 | 0.032 |
| DN | 0.146 | 0.040 | 3.674 | <0.001 |
| HII | -0.045 | 0.054 | -0.834 | 0.404 |
| AP | -0.352 | 0.074 | -4.774 | <0.001 |
| AMW | 0.101 | 0.041 | 2.478 | 0.013 |
| PD | 0.067 | 0.055 | 1.225 | 0.221 |
| **Tree** |  |  |  |  |
| A | 0.317 | 0.165 | 1.924 | 0.054 |
| DM | -0.295 | 0.214 | -1.379 | 0.168 |
| DN | 0.117 | 0.117 | 1.003 | 0.316 |
| HII | -0.091 | 0.162 | -0.559 | 0.576 |
| AP | -0.169 | 0.223 | -0.756 | 0.450 |
| AMW | 0.106 | 0.117 | 0.907 | 0.365 |
| PD | 0.100 | 0.160 | 0.627 | 0.531 |
| **Shrub** |  |  |  |  |
| A | 0.384 | 0.142 | 2.702 | 0.007 |
| DM | -0.057 | 0.169 | -0.340 | 0.734 |
| DN | 0.268 | 0.092 | 2.913 | 0.004 |
| HII | 0.157 | 0.128 | 1.227 | 0.220 |
| AP | -0.415 | 0.168 | -2.473 | 0.013 |
| AMW | -0.001 | 0.099 | -0.013 | 0.990 |
| PD | 0.133 | 0.125 | 1.057 | 0.291 |
| **Herb** |  |  |  |  |
| A | 0.426 | 0.068 | 6.265 | <0.001 |
| DM | -0.148 | 0.088 | -1.674 | 0.094 |
| DN | 0.123 | 0.048 | 2.548 | 0.011 |
| HII | -0.084 | 0.064 | -1.308 | 0.191 |
| AP | -0.360 | 0.089 | -4.065 | <0.001 |
| AMW | 0.128 | 0.048 | 2.645 | 0.008 |
| PD | 0.037 | 0.066 | 0.566 | 0.571 |
| **Invasive plants** |  |  |  |  |
| A | 0.557 | 0.206 | 2.704 | 0.007 |
| DM | -0.602 | 0.321 | -1.877 | 0.061 |
| DN | 0.154 | 0.142 | 1.092 | 0.275 |
| HII | 0.019 | 0.190 | 0.101 | 0.919 |
| AP | -0.726 | 0.297 | -2.44 | 0.015 |
| AMW | 0.155 | 0.139 | 1.114 | 0.265 |
| PD | 0.353 | 0.224 | 1.578 | 0.115 |

**Table S7.** Generalised linear modelling assesses the impact and significance of various environmental factors on Shannon-Wiener diversity index.

|  | **Estimate** | **Std.Error** | **z-value** | **Pr(>\|z\|)** |
| --- | --- | --- | --- | --- |
| **All plants** |  |  |  |  |
| A | 0.156 | 0.105 | 1.491 | 0.274 |
| DM | -0.251 | 0.103 | -2.436 | 0.135 |
| DN | 0.098 | 0.075 | 1.309 | 0.321 |
| HII | -0.199 | 0.096 | -2.071 | 0.174 |
| AP | -0.235 | 0.115 | -2.043 | 0.178 |
| AMW | -0.110 | 0.076 | -1.447 | 0.285 |
| PD | 0.034 | 0.081 | 0.413 | 0.719 |
| **Tree** |  |  |  |  |
| A | 0.436 | 0.341 | 1.280 | 0.329 |
| DM | -1.097 | 0.335 | -3.274 | 0.082 |
| DN | 0.099 | 0.245 | 0.405 | 0.725 |
| HII | -0.221 | 0.313 | -0.704 | 0.554 |
| AP | -0.808 | 0.375 | -2.153 | 0.164 |
| AMW | 0.049 | 0.247 | 0.197 | 0.862 |
| PD | 0.530 | 0.265 | 1.999 | 0.184 |
| **Shrub** |  |  |  |  |
| A | 0.144 | 0.030 | 4.780 | 0.041 |
| DM | -0.032 | 0.030 | -1.093 | 0.389 |
| DN | 0.032 | 0.022 | 1.475 | 0.278 |
| HII | -0.015 | 0.028 | -0.526 | 0.652 |
| AP | -0.088 | 0.033 | -2.659 | 0.117 |
| AMW | -0.153 | 0.022 | -7.008 | 0.020 |
| PD | 0.050 | 0.023 | 2.143 | 0.165 |
| **Herb** |  |  |  |  |
| A | 0.212 | 0.117 | 1.819 | 0.210 |
| DM | -0.299 | 0.115 | -2.606 | 0.121 |
| DN | 0.138 | 0.084 | 1.649 | 0.241 |
| HII | -0.224 | 0.107 | -2.086 | 0.172 |
| AP | -0.318 | 0.128 | -2.479 | 0.131 |
| AMW | -0.114 | 0.085 | -1.349 | 0.310 |
| PD | 0.054 | 0.091 | 0.590 | 0.615 |

**Table S8.** Generalised linear modelling assesses the impact and significance of various environmental factors on Simpson diversity index.

|  | **Estimate** | **Std.Error** | **z-value** | **Pr(>\|z\|)** |
| --- | --- | --- | --- | --- |
| **All plants** |  |  |  |  |
| A | 0.089 | 0.092 | 0.968 | 0.435 |
| DM | -0.164 | 0.091 | -1.81 | 0.212 |
| DN | 0.079 | 0.066 | 1.195 | 0.355 |
| HII | -0.174 | 0.085 | -2.050 | 0.177 |
| AP | -0.143 | 0.102 | -1.402 | 0.296 |
| AMW | -0.085 | 0.067 | -1.265 | 0.333 |
| PD | 0.012 | 0.072 | 0.164 | 0.884 |
| **Tree** |  |  |  |  |
| A | 0.481 | 0.361 | 1.334 | 0.314 |
| DM | -1.32 | 0.354 | -3.725 | 0.065 |
| DN | 0.163 | 0.259 | 0.629 | 0.594 |
| HII | -0.224 | 0.331 | -0.677 | 0.568 |
| AP | -0.964 | 0.397 | -2.428 | 0.136 |
| AMW | 0.077 | 0.262 | 0.295 | 0.796 |
| PD | 0.689 | 0.281 | 2.455 | 0.134 |
| **Shrub** |  |  |  |  |
| A | 0.112 | 0.026 | 4.350 | 0.049 |
| DM | -0.016 | 0.025 | -0.619 | 0.599 |
| DN | -0.010 | 0.018 | -0.517 | 0.657 |
| HII | -0.025 | 0.024 | -1.050 | 0.404 |
| AP | -0.056 | 0.028 | -1.964 | 0.188 |
| AMW | -0.115 | 0.019 | -6.157 | 0.025 |
| PD | 0.043 | 0.020 | 2.166 | 0.163 |
| **Herb** |  |  |  |  |
| A | 0.137 | 0.130 | 1.054 | 0.402 |
| DM | -0.190 | 0.128 | -1.488 | 0.275 |
| DN | 0.113 | 0.093 | 1.214 | 0.349 |
| HII | -0.205 | 0.119 | -1.717 | 0.228 |
| AP | -0.200 | 0.143 | -1.401 | 0.296 |
| AMW | -0.109 | 0.094 | -1.155 | 0.368 |
| PD | 0.011 | 0.101 | 0.112 | 0.921 |

**Table S9.** Generalised linear modelling assesses the impact and significance of various environmental factors on Pielou evenness index.

|  | **Estimate** | **Std.Error** | **z-value** | **Pr(>\|z\|)** |
| --- | --- | --- | --- | --- |
| **All plants** |  |  |  |  |
| A | 0.093 | 0.091 | 1.020 | 0.415 |
| DM | -0.216 | 0.090 | -2.404 | 0.138 |
| DN | 0.067 | 0.066 | 1.020 | 0.415 |
| HII | -0.188 | 0.084 | -2.241 | 0.154 |
| AP | -0.162 | 0.101 | -1.612 | 0.248 |
| AMW | -0.110 | 0.066 | -1.663 | 0.238 |
| PD | 0.019 | 0.071 | 0.261 | 0.819 |
| **Tree** |  |  |  |  |
| A | 0.281 | 0.271 | 1.037 | 0.409 |
| DM | -0.943 | 0.266 | -3.543 | 0.071 |
| DN | 0.097 | 0.195 | 0.496 | 0.669 |
| HII | -0.159 | 0.249 | -0.640 | 0.588 |
| AP | -0.693 | 0.298 | -2.322 | 0.146 |
| AMW | 0.040 | 0.197 | 0.203 | 0.858 |
| PD | 0.470 | 0.211 | 2.227 | 0.156 |
| **Shrub** |  |  |  |  |
| A | 0.098 | 0.084 | 1.164 | 0.364 |
| DM | -0.046 | 0.082 | -0.553 | 0.636 |
| DN | -0.014 | 0.060 | -0.225 | 0.843 |
| HII | 0.003 | 0.077 | 0.040 | 0.972 |
| AP | -0.090 | 0.092 | -0.974 | 0.433 |
| AMW | -0.147 | 0.061 | -2.412 | 0.137 |
| PD | 0.076 | 0.065 | 1.172 | 0.362 |
| **Herb** |  |  |  |  |
| A | 0.127 | 0.101 | 1.261 | 0.335 |
| DM | -0.230 | 0.099 | -2.315 | 0.147 |
| DN | 0.094 | 0.073 | 1.299 | 0.324 |
| HII | -0.215 | 0.093 | -2.317 | 0.146 |
| AP | -0.198 | 0.111 | -1.777 | 0.218 |
| AMW | -0.121 | 0.073 | -1.651 | 0.241 |
| PD | 0.024 | 0.079 | 0.302 | 0.791 |

**Table S10.** Redundancy analyses assessed the effects and significance of environmental variables on plant distribution.

| Factor | All plants | | Tree | | Shrub | | Herb | | Invasive plants | |
| --- | --- | --- | --- | --- | --- | --- | --- | --- | --- | --- |
|  | r2 | p-value | r2 | p-value | r2 | p-value | r2 | p-value | r2 | p-value |
| A | 0.856 | 0.003 | 0.789 | 0.006 | 0.756 | 0.008 | 0.732 | 0.008 | 0.906 | 0.001 |
| DM | 0.806 | 0.005 | 0.546 | 0.039 | 0.530 | 0.050 | 0.827 | 0.002 | 0.521 | 0.066 |
| DN | 0.417 | 0.111 | 0.469 | 0.193 | 0.616 | 0.110 | 0.224 | 0.445 | 0.148 | 0.533 |
| HII | 0.519 | 0.059 | 0.733 | 0.012 | 0.704 | 0.013 | 0.409 | 0.141 | 0.673 | 0.016 |
| AP | 0.503 | 0.096 | 0.571 | 0.039 | 0.660 | 0.015 | 0.459 | 0.125 | 0.572 | 0.053 |
| AMW | 0.387 | 0.174 | 0.166 | 0.533 | 0.346 | 0.220 | 0.392 | 0.170 | 0.805 | 0.004 |
| PD | 0.261 | 0.358 | 0.133 | 0.629 | 0.183 | 0.498 | 0.423 | 0.159 | 0.154 | 0.547 |
